# A Robust, Highly Multiplexed Mass Spectrometry Assay to Identify SARS-CoV-2 Variants

**DOI:** 10.1128/spectrum.01736-22

**Published:** 2022-09-07

**Authors:** Matthew M. Hernandez, Radhika Banu, Paras Shrestha, Ana S. Gonzalez-Reiche, Adriana van de Guchte, Keith Farrugia, Robert Sebra, Mount Sinai PSP Study Group, Melissa R. Gitman, Michael D. Nowak, Carlos Cordon-Cardo, Viviana Simon, Harm van Bakel, Emilia Mia Sordillo, Nicolas Luna, Angie Ramirez, Sergio Andres Castañeda, Luz Helena Patiño, Nathalia Ballesteros, Marina Muñoz, Juan David Ramírez, Alberto E. Paniz-Mondolfi

**Affiliations:** a Department of Pathology, Molecular, and Cell-Based Medicine, Icahn School of Medicine at Mount Sinai, New York, New York, USA; b Department of Genetics and Genomic Sciences, Icahn School of Medicine at Mount Sinai, New York, New York, USA; c Icahn Genomics Institute, Icahn School of Medicine at Mount Sinai, New York, New York, USA; d Black Family Stem Cell Institute, Icahn School of Medicine at Mount Sinai, New York, New York, USA; e Sema4, a Mount Sinai venture, Stamford, Connecticut, USA; f Department of Microbiology, Icahn School of Medicine at Mount Sinai, New York, New York, USA; g Center for Vaccine Research and Pandemic Preparedness (C-VARPP), Icahn School of Medicine at Mount Sinai, New York, New York, USA; h Division of Infectious Diseases, Department of Medicine, Icahn School of Medicine at Mount Sinai, New York, New York, USA; i The Global Health and Emerging Pathogens Institute, Icahn School of Medicine at Mount Sinai, New York, New York, USA; j Centro de Investigaciones en Microbiología y Biotecnología-UR (CIMBIUR), Facultad de Ciencias Naturales, Universidad del Rosario, Bogotá, Colombia; University of Cincinnati

**Keywords:** RT-PCR, MALDI-TOF, SARS-CoV-2, variant panel, multiplex, Omicron

## Abstract

Severe acute respiratory syndrome coronavirus 2 (SARS-CoV-2) variants are characterized by differences in transmissibility and response to therapeutics. Therefore, discriminating among them is vital for surveillance, infection prevention, and patient care. While whole-genome sequencing (WGS) is the “gold standard” for variant identification, molecular variant panels have become increasingly available. Most, however, are based on limited targets and have not undergone comprehensive evaluation. We assessed the diagnostic performance of the highly multiplexed Agena MassARRAY SARS-CoV-2 Variant Panel v3 to identify variants in a diverse set of 391 SARS-CoV-2 clinical RNA specimens collected across our health systems in New York City, USA and Bogotá, Colombia (September 2, 2020 to March 2, 2022). We demonstrated almost perfect levels of interrater agreement between this assay and WGS for 9 of 11 variant calls (κ ≥ 0.856) and 25 of 30 targets (κ ≥ 0.820) tested on the panel. The assay had a high diagnostic sensitivity (≥93.67%) for contemporary variants (e.g., Iota, Alpha, Delta, and Omicron [BA.1 sublineage]) and a high diagnostic specificity for all 11 variants (≥96.15%) and all 30 targets (≥94.34%) tested. Moreover, we highlighted distinct target patterns that could be utilized to identify variants not yet defined on the panel, including the Omicron BA.2 and other sublineages. These findings exemplified the power of highly multiplexed diagnostic panels to accurately call variants and the potential for target result signatures to elucidate new ones.

**IMPORTANCE** The continued circulation of SARS-CoV-2 amid limited surveillance efforts and inconsistent vaccination of populations has resulted in the emergence of variants that uniquely impact public health systems. Thus, in conjunction with functional and clinical studies, continuous detection and identification are quintessential to informing diagnostic and public health measures. Furthermore, until WGS becomes more accessible in the clinical microbiology laboratory, the ideal assay for identifying variants must be robust, provide high resolution, and be adaptable to the evolving nature of viruses like SARS-CoV-2. Here, we highlighted the diagnostic capabilities of a highly multiplexed commercial assay to identify diverse SARS-CoV-2 lineages that circulated from September 2, 2020 to March 2, 2022 among patients seeking care in our health systems. This assay demonstrated variant-specific signatures of nucleotide/amino acid polymorphisms and underscored its utility for the detection of contemporary and emerging SARS-CoV-2 variants of concern.

## INTRODUCTION

Since the onset of the coronavirus disease 2019 (COVID-19) pandemic, suboptimal surveillance and diagnostic efforts have not been able to prevent the rapid, unchecked spread of severe acute respiratory syndrome coronavirus 2 (SARS-CoV-2) (1–4). In conjunction with various factors (e.g., variable health care access, limitations to effective infection prevention efforts), continued spread has led to the emergence of viral variants characterized by increased genomic diversity, including the most recent Omicron (B.1.1.529) variant and its sublineages (5–8). This poses a unique challenge to health care systems and diagnostic laboratories alike because genomic variation has the potential to impact viral fitness (5, 9), disease pathogenesis (10–12), response to therapeutics (e.g., antibodies) (5, 13, 14), and molecular target detection (15–17).

Ideally, SARS-CoV-2 diagnostic assays should be scalable to test increased clinical specimens and should be robust enough to accommodate genomic variation in viruses over time. Although improved technologies have made high-throughput platforms more available, most are limited in the level of multiplexing and, thus, risk target dropout and failure to capture infected individuals. Indeed, current nucleic acid amplification tests (e.g., reverse-transcription PCR [RT-PCR]) largely utilize 1 to 3 targets to detect (e.g., presence/absence) SARS-CoV-2 nucleic acids. Moreover, as new variants have emerged, diagnostic panels are based on targets designed for the detection of nucleotide changes that yield specific amino acid substitutions and call variants based on distinct target result combinations (18–20). However, most of these assays distinguish viral variants through result patterns of 3 to 9 molecular targets across multiple reaction wells (20–28), which are constrained to distinguishing current circulating variants but may not be sufficient to distinguish nascent, increasingly divergent variants.

Whole-genome sequencing (WGS) has, therefore, largely served as the “gold standard” for pathogen genomic surveillance. Still, this methodology is not realistic for most diagnostic laboratories because it requires staff with bioinformatic expertise and infrastructure, is relatively expensive, and is restricted in lower-income countries (LICs) and lower-middle-income countries (LMCs) (29, 30). Therefore, there is great potential for highly multiplexed assays that target an expansive repertoire of polymorphisms. Currently, these platforms are rare in number (31, 32) and most have not yet been evaluated for their diagnostic capabilities in the clinical setting.

Here, we recovered 391 SARS-CoV-2 viral RNA from clinical specimens collected from infected individuals who presented for testing at the Mount Sinai Health System (MSHS) in New York City (NYC) and the Universidad del Rosario in Bogotá, Colombia from September 2, 2020 to March 2, 2022. These specimens had previously undergone WGS for epidemiologic surveillance, and we used these data as a benchmark to evaluate the diagnostic performance of the Agena MassARRAY SARS-CoV-2 Variant Panel v3 (research use only, RUO), which utilizes mass spectrometry to detect target products amplified from viral RNA by RT-PCR. We tested this highly diverse set of viral variants to interrogate the level of agreement and diagnostic sensitivity and specificity across 12 distinct variants on the panel and 30 distinct polymorphic targets in the *Spike* (S) gene region. We demonstrated a high level of assay agreement and high levels of diagnostic sensitivity and specificity across most variant and individual targets tested. Furthermore, we highlighted the utility of the variant panel to elucidate undefined or emergent variants based on unique target result signatures.

## RESULTS

**FIG 1:**
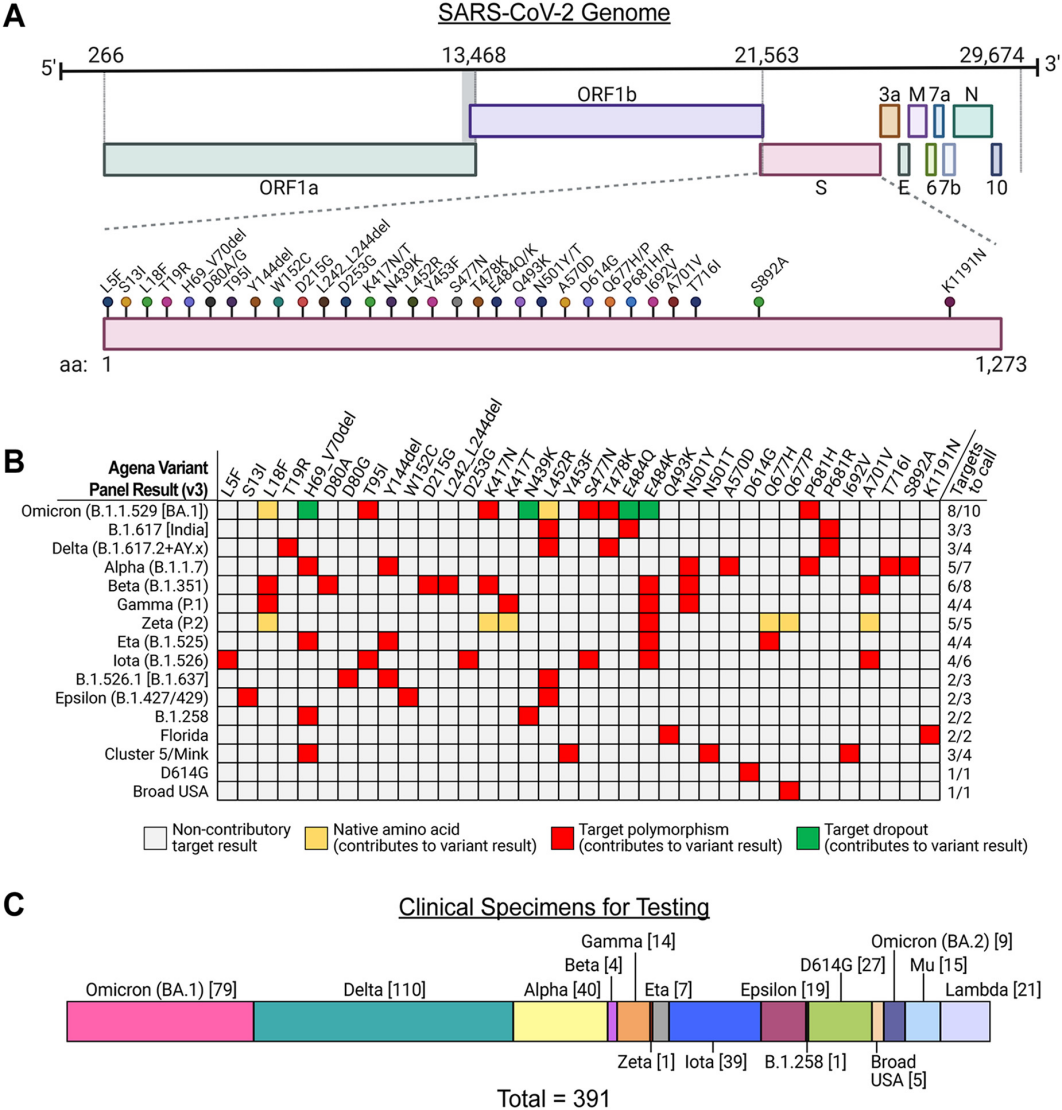
Detection of viral variants by the Agena MassARRAY SARS-CoV-2 Variant Panel. (A) SARS-CoV-2 genome with nucleotide positions from 5′-to-3′ direction depicted above. *S* gene polymorphisms targeted by the variant panel (lollipops) and corresponding amino acids are depicted below. (B) A color map depicts algorithms of target combinations that defined 16 distinct SARS-CoV-2 variants on the panel. Variant results are depicted (left) and included the WHO designation (e.g., Omicron, Delta, etc.) and corresponding PANGO lineage assignments. Note that the B.1.526.1 variant was redesignated B.1.637 to distinguish it from the Iota variant lineage (https://cov-lineages.org/lineage_list.html, accessed April 26, 2022). The minimum number of targets required to support the corresponding variant result is indicated (right). Target results are depicted as colored cells indicating amino acid positions that did not contribute to the defined variant identity algorithm (gray). The remaining three colors reflect native amino acids (e.g., unchanged from Wuhan-Hu-1 reference) (yellow), detectable amino acid polymorphisms (red), and drop out of the given target (green), all of which contributed to the variant identity algorithm. (C) Phylogenetic composition of 391 clinical specimen viral RNA recovered for diagnostic evaluation of the variant panel. The numbers of each lineage tested are depicted in brackets.

We analyzed a diverse set of 391 SARS-CoV-2 viral RNA specimens that were collected from infected patients over 18 months of the COVID-19 pandemic in the NYC metropolitan area and Colombia. This RNA all underwent WGS that resulted in consensus genomes that comprise 56 distinct phylogenetic PANGO lineages and corresponded to 12 of the 16 possible variant calls on the panel (Fig. 1C). These included 39 Iota (B.1.526), 40 Alpha (B.1.1.7), 110 Delta (B.1.617.2 (*n* = 3) + AY.x [*n* = 107]), and 79 Omicron (B.1.1.529 [BA.1 sublineage]) specimens. We also included 45 specimens that corresponded to 3 variants that were not defined by the panel (e.g., Lambda [C.37], Mu [B.1.621], Omicron [BA.2 sublineage]) to interrogate the assay’s ability to distinguish these based on target result patterns.

### Diagnostic performance of variant calling.

To evaluate the diagnostic performance of the Agena MassARRAY Variant Panel, phylogenetic results of consensus sequences based on WGS data served as the “gold standard” for comparison. Of 391 specimens tested on the variant panel, there were 62 with variant calls that were discordant with WGS data. However, 45 of these consisted of specimens whose sequenced variant was without an appropriately defined variant algorithm on the panel. Therefore, only 17 (4.91%) of clinical RNA tested yielded discordant results between WGS and the expected results on the variant panel.

We measured the level of agreement between the overall variant calls from WGS and the variant panel (Table 1). Of the 12 panel variants in our study set, we performed agreement analyses on 11. We could not measure the level of agreement – or diagnostic sensitivity/specificity – for the isolated D614G result because specimens that concurrently encoded the native D614 amino acid and did not yield any other variant result were not recovered for this study.

**TABLE 1:** Diagnostic agreement between WGS and panel variant calls

Variant	No. of Specimens^*a*^	Agreement (%)	Kappa (95% CI)	Interpretation^*b*^
Omicron (BA.1)	79	98.72 %	0.959 (0.924–0.995)	Almost perfect
Delta	110	98.72 %	0.968 (0.940–0.996)	Almost perfect
Alpha	40	100.00 %	1.000 (1.000–1.000)	Almost perfect
Beta	4	99.74 %	0.856 (0.577–1.000)	Almost perfect
Gamma	14	99.74 %	0.964 (0.894–1.000)	Almost perfect
Zeta	1	95.91 %	−0.005 (−0.014–0.004)	None
Eta	7	98.47 %	0.247 (0.147–0.640)	Fair
Iota	39	99.74 %	0.986 (0.957–1.000)	Almost perfect
Epsilon	19	100.00 %	1.000 (1.000–1.000)	Almost perfect
B.1.258	1	100.00 %	1.000 (1.000–1.000)	Almost perfect
D614G^*c*^	27	NA	NA	NA
Broad USA	5	100.00 %	1.000 (1.000–1.000)	Almost perfect

a Number of specimens confirmed by WGS as the indicated variant.

b Interpretation of the level of agreement is based on reference (49).

c Agreement analyses were not performed because specimens that harbored the native D614 amino acid were not recovered for testing. NA, not available.

Overall, we observed a high level of agreement (e.g., κ ≥ 0.856) for 9/11 variants, including the contemporary Delta (B.1.617.2+AY.x) and Omicron (BA.1) variants. The Zeta (P.2) and Eta (B.1.525) variant calls demonstrated the lowest level of agreement. The single Zeta variant confirmed by WGS that was tested (PV26936) resulted in the Florida variant on the panel (Table S4 in Supplemental File 1). Interestingly, although the E484K and the four native amino acids – L18, K417, A701, and Q677H – were correctly detected as part of the Zeta target algorithm, the detection of K1191N and Q493K targets met the Florida variant target result criteria. The low level of agreement for the Zeta variant call was also impacted by the fact that all 15 Mu (B.1.621) specimens tested on the panel were incorrectly identified as Zeta. However, it is important to note that the Mu variant was not yet defined on this panel. Of the 7 Eta (B.1.525) variants tested on the panel, only 1 correctly identified as Eta while the remaining 6 resulted as detected D614G. These did not meet the minimum number of detectable targets to yield the Eta result and only resulted in 2 to 3 of the 4 minimum required targets. These results may be the consequences of RNA degradation over long-term storage. For example, the six discordant specimens encoded the H69_V70del by WGS but all yielded drop out of that target on the panel, which further supported this scenario.

**FIG 2:**
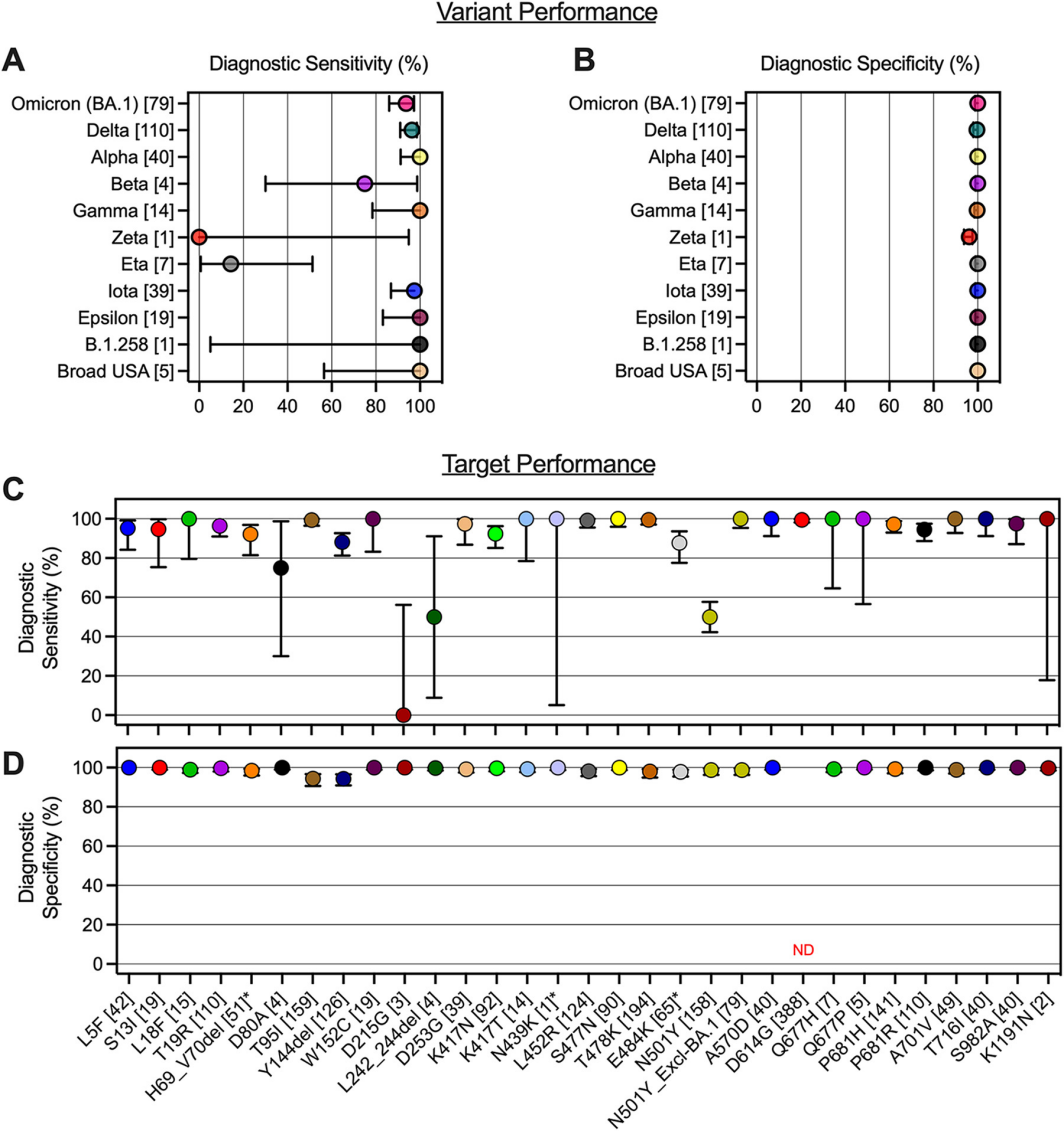
Diagnostic sensitivity and specificity of the Agena MassARRAY SARS-CoV-2 Variant panel. (A) Diagnostic sensitivity and (B) diagnostic specificity of 11 variant calls on the panel are depicted. The number of specimens that correspond with each variant according to WGS is annotated in brackets. Depiction of (C) diagnostic sensitivity and (D) diagnostic specificity of each of 30 distinct panel targets. The number of specimens that correspond with each amino acid polymorphism according to WGS is annotated in brackets for each target. Asterisks (*) indicate targets for which dropout results were excluded from analyses (see Materials and Methods). For target N501Y, a separate diagnostic analysis was conducted excluding BA.1 specimens (“N501Y_Excl-BA.1”). Error bars reflect 95% CI in all four panels. ND, not determined.

We also measured the diagnostic sensitivity and specificity of the panel (Fig. 2A and B) for the variants tested. Diagnostic sensitivity ranged from 0% (95% CI, 0 to 94.87%; Zeta) to 100% (95% CI, 91.24 to 100%; Alpha). Unsurprisingly, the specimens for which we had limited (e.g., <10) specimens available for testing had the lowest measured sensitivities and broadest CIs, including Zeta and Eta variants. Among variants for which we recovered >10 specimens for testing, diagnostic sensitivity was ≥93.67% (Omicron [BA.1]). In addition, the variant panel demonstrated a high level of diagnostic specificity across all 11 variants tested ranging from 96.15% (95% CI, 93.75 to 97.66%) for the Zeta variant to >99% for all other variants (Fig. 2B). Furthermore, excluding the Zeta variant, the panel results showed high PPVs (≥0.933) and high NPVs (≥0.984) for all variant calls (Table S5 in Supplemental File 1).

### Diagnostic performance of distinct target calls.

To evaluate the diagnostic capabilities of each of the 36 targeted polymorphisms that comprise the variant panel, we performed interrater agreement analyses and measured the diagnostic sensitivity and specificity of each assay target. Across all 391 viral RNA specimens, each of 30 of the possible 36 polymorphisms was present in at least one specimen.

When we performed agreement analyses on each of these 30 targets (Table 2), 25 demonstrated almost perfect levels of agreement (κ ≥ 0.820). The targets with suboptimal levels of agreement for our data set included D215G (κ = 0; no agreement), L242_244del (κ = 0.568; moderate agreement), N501Y (κ = 0.528; moderate agreement), and K1191N (κ = 0.799; substantial agreement). This low level of agreement may be impacted by the small sample sizes tested. Indeed, we only recovered 2 to 4 specimens that encoded each of the D215G, L242_244del, and K1191N targets. Therefore, small frequencies (e.g., 2 to 4) of inaccurate calls may explain this result. It is important to note that our study set did not include specimens with the native D614 amino acid (A24303 nucleotide), and the level of agreement could not be calculated for the D614G target.

**TABLE 2:** Diagnostic agreement between WGS and panel target calls

Target	No. of Specimens^*a*^^,^^*b*^	Agreement (%)	Kappa (95% CI)	Interpretation^*c*^
L5F	42	99.49 %	0.973 (0.935–1.000)	Almost perfect
S13I	19	99.74 %	0.972 (0.916–1.000)	Almost perfect
L18F	15	98.99 %	0.877 (0.758–0.996)	Almost perfect
T19R	110	98.72 %	0.968 (0.940–0.996)	Almost perfect
H69_V70del	51	97.35 %	0.906 (0.841–0.970)	Almost perfect
D80A	4	99.74 %	0.856 (0.577–1.000)	Almost perfect
D80G	0	NA	NA	NA
T95I	159	96.42 %	0.927 (0.889–0.964)	Almost perfect
Y144del	126	92.33 %	0.824 (0.764–0.885)	Almost perfect
W152C	19	100.00 %	1.000 (1.000–1.000)	Almost perfect
D215G	3	99.23 %	0	None
L242_244del	4	99.23 %	0.568 (0.127–1.000)	Moderate
D253G	39	98.98 %	0.944 (0.890–0.999)	Almost perfect
K417N	92	97.95 %	0.942 (0.902–0.982)	Almost perfect
K417T	14	99.23 %	0.899 (0.786–1.000)	Almost perfect
N439K	1	100.00 %	1.000 (1.000–1.000)	Almost perfect
L452R	124	98.47 %	0.965 (0.937–0.933)	Almost perfect
Y453F	0	NA	NA	NA
S477N	90	100.00 %	1.000 (1.000–1.000)	Almost perfect
T478K	194	98.72 %	0.974 (0.952–0.997)	Almost perfect
E484Q	0	NA	NA	NA
E484K	65	95.79 %	0.860 (0.791–0.929)	Almost perfect
Q493K	0	NA	NA	NA
N501Y	158	79.03 %	0.528 (0.447–0.609)	Moderate
N501Y (exclude BA.1)	79	99.04 %	0.975 (0.947–1.000)	Almost perfect
N501T	0	NA	NA	NA
A570D	40	100.00 %	1.000 (1.000–1.000)	Almost perfect
D614G^*d*^	388	99.49 %	NA	NA
Q677H	7	99.23 %	0.820 (0.620–1.000)	Almost perfect
Q677P	5	100.00 %	1.000 (1.000–1.000)	Almost perfect
P681H	141	98.47 %	0.967 (0.940–0.993)	Almost perfect
P681R	110	98.47 %	0.961 (0.931–0.992)	Almost perfect
I692V	0	NA	NA	NA
A701V	49	98.88 %	0.955 (0.911–0.999)	Almost perfect
T716I	40	100.00 %	1.000 (1.000–1.000)	Almost perfect
S982A	40	99.74 %	0.986 (0.958–1.000)	Almost perfect
K1191N	2	99.74 %	0.799 (0.413–1.000)	Substantial

a Number of specimens that harbored the given target polymorphism by WGS.

b Analyses were not performed if specimens with the given target polymorphism by WGS were not recovered for testing. NA, not available.

c Interpretation of the level of agreement is based on reference (49).

d Kappa could not be determined because specimens that harbored the native D614 amino acid were not recovered for testing.

Interestingly, for the N501Y target, we found that of 158 specimens with the polymorphism by WGS, 79 yielded a false-negative result on the variant panel. All 79 belong to the Omicron (BA.1) variant lineage, and when reanalyzed excluding these BA.1 specimens, the interrater agreement was almost perfect (κ = 0.975) (Table 2). This suggested genomic variation outside the original assay design and within a given lineage may impact primer/probe binding and yield distinct target results for novel variants.

Across the 30 targets tested, the average diagnostic sensitivity measured was 90.2% (Fig. 2C). The targets with the lowest sensitivities included D80A (75%; 95% CI, 30.06% to 98.72%), D215G (0%; 95% CI, 0 to% 56%), L242_244del (50%; 95% CI, 9% to 91%), and N501Y (50%; 95% CI, 42% to 58%). Notably, when all BA.1 specimens were excluded from the analyses, the sensitivity of the N501Y target improved to 100.00% (95% CI, 95% to 100%).

The variant panel assay demonstrated a high diagnostic specificity across nearly all 30 targets tested in this study (Fig. 2D). On average, the diagnostic specificity was >99% across all the tested diagnostic targets. Specificity was not calculated for the D614G target because no clinical specimens with the native D614 amino acid were recovered in this study. In addition, across the 30 targets, the PPVs and NPVs were 0.9 and 0.959, respectively (Table S6 in Supplemental File 1).

### Diagnostic target signatures of undefined variants.

**FIG 3:**
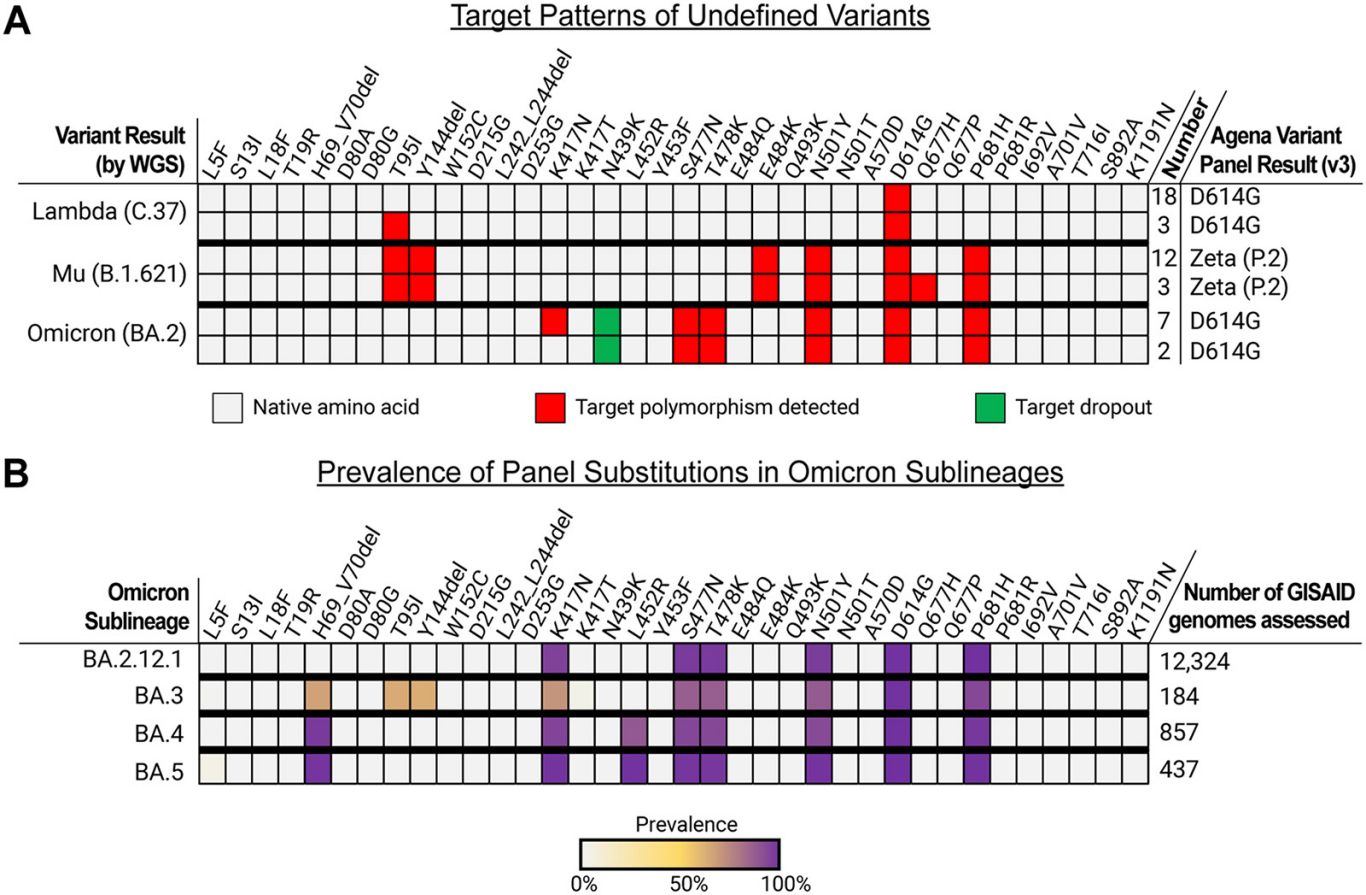
Target result patterns of undefined variants on the Agena MassARRAY SARS-CoV-2 Variant Panel. (A) A color map depicts the observed target results for three undefined SARS-CoV-2 variants tested on the panel: Lambda (C.37), Mu (B.1.621), and Omicron (BA.2). Distinct target patterns were observed among each of the variant types are depicted. Cells indicate the distinct target results, including detectable native amino acid (gray), detection of target polymorphism (red), and target dropout (green). The number of specimens that yielded each of the distinct target result patterns is indicated on the right as well as the output variant ID result generated by the variant panel software. (B) A heatmap depicts the measured prevalence of each variant panel target substitution among publicly available Omicron sublineage genomes as of May 6, 2022.

Given that the variant panel has a uniquely high level of multiplexing, we also interrogated the capabilities of the assay to reveal unique signatures of variants not defined by the panel software. To do this, we included 45 clinical specimens that included the older Lambda (C.37) (*n* = 21) and Mu (B.1.621) (*n* = 15) variants as well as the contemporary Omicron BA.2 variants (*n* = 9) recently captured in NYC (Fig. 3A). Each of the three was called as D614G, Zeta (P.2), and D614G, respectively, on the panel.

Based on the current design of the panel, most of the Lambda specimens tested (18/21) only have detectable D614G polymorphisms among the 36 targets. The remaining 3 additionally yield a T95I (C21846U) polymorphism. However, this substitution was not found in any of the 3 consensus sequences and, therefore, may represent a nonspecific reaction or a minority intrahost variant. Notably, this polymorphism was rare and found in only 24/10186 (0.23%) Lambda genomes deposited in Global Initiative on Sharing Avian Influenza Data (GISAID) database (www.gisaid.org, last accessed May 6, 2022). The current target design did not target common Lambda substitutions, including G75V, T76I, D253N, L452Q, T859N, or deletions (e.g., amino acids 246 to 252).

All 15 Mu specimens were appropriately called Zeta (P.2) based on the presence of the native L18, K417, Q677, and A701 amino acids as well as the E484K that met the threshold for the Zeta variant call. All Mu specimens display a signature of six detectable targets that was unique among all other variant patterns on this assay: T95I, Y144del, E484K, N501Y, D614G, and P681H. Interestingly, none of the specimens’ consensus genome sequences encoded the Y144del, which suggested other sequence variations may alter primer/probe binding to this target. Twelve of the Mu sequences harbored substitutions at positions 144 to 145 (e.g., Y144S, Y145N), which may have impacted target detection. The five other amino acid substitutions were encoded in the consensus genomes of all 15 specimens. Of note, although specimen K42 featured an adenosine insertion at genome position 21995, the downstream nucleotide sequence encodes these amino acid polymorphisms. In addition to these 5 substitutions, 3 Mu specimens yielded a detectable Q677H target. However, these genomes harbored the G23593 nucleotide, which encoded the native Q677 amino acid, and suggested this was a nonspecific result or detection of a minor intrahost variant.

We also found that the 9 Omicron BA.2 specimens generated a distinct target result signature on the variant panel assay. All 9 resulted in the detection of S477N, T478K, N501Y, D614G, and P681H targets as well as the dropout of the N439K target. The five detected targets each were confirmed by the presence of the amino acid substitutions in WGS data. We cannot delineate the cause of the N439K target dropout because we do not know primer/probe sequences at the site of the targeted nucleotide substitution at position 22879. However, one can speculate that sequence variation around this region may interfere with primer/probe binding. Indeed, all 9 of the BA.2 specimens harbored the T22882G polymorphism, which resulted in the N440K substitution. Furthermore, the K417N substitution was found in all BA.2 consensus genomes but was only detected in 7 specimens. This may have been the result of different nucleic acid quantities across specimens and reflected a limit in analytic sensitivity for the target. Together, these were important findings to gauge the capabilities of this assay to highlight unique target signatures of variants that might be captured by this platform.

Finally, to assess the capabilities of the assay to detect other emergent Omicron sublineages (e.g., BA.2.12.1, BA.3, BA.4, and BA.5), we independently interrogated the prevalence of each variant panel target substitution among publicly available genomes (GISAID) (Fig. 3B). We found that 94.9 to 99.7% of BA.2.12.1 genomes harbored each of the K417N, S477N, T478K, N501Y, D614G, and P681H substitutions. Except for the N439K target dropout in BA.2 genomes tested in this study, the BA.2.12.1 target signature harbored the same detectable polymorphisms. While 70.7 to 99.5% of BA.3 genomes also encoded the S477N, T478, N501Y, D614G, and P681H; 63.0 to 66.3% harbored the H69_V70del, T95I, and Y144del substitution, which may distinguish this sublineage from BA.2 and BA.2.12.1. Furthermore, 88.1% of BA.4 and 99.3% of BA.5 genomes encoded the L452R substitution, which can help to differentiate these genomes from other Omicron sublineages.

Given these *in silico* findings, it was important to consider these polymorphisms in the context of genomic variation that may occur at proprietary primer/probe binding sites. Indeed, a review of the publicly available alignment of representative forms of Spike variants (from Los Alamos National Laboratory (33); https://www.epicov.org/epi3/frontend#7379e, accessed June 30, 2022), revealed distinct polymorphisms in the Omicron sublineage variants that may have affected target performance. For example, A67V (C21762T), G142D (G21987A and T21988C), and G446S (G22898A) were harbored by BA.1 and BA.3, but not BA.2, BA.4, and BA.5, representative genomes. While not targeted directly by the variant panel, their proximity to targeted codons 69 to 70, 95, 417, and 452 may have resulted in target dropout and shaping of BA.1 and BA.3 target result signatures. The same could be said for G142D (G21987A) and R408S (A22786T), which were found in BA.2, BA.4, and BA.5 but not BA.1 or BA.3 representative genomes. These considerations warrant further testing because these variants emerged and further highlighted the utility of future diagnostic evaluation.

## DISCUSSION

Monoclonal antibody treatments are effective in limiting severe COVID-19 but emerging variants of concern often carry mutations that render the virus partially or completely resistant to antibody neutralization (13, 14, 34–38). Rapid SARS-CoV-2 variant calling is, therefore, essential for personalized COVID-19 treatment interventions. With the advent of commercial and lab-developed variant panels, however, accurate variant calling requires robust, high-resolution platforms that are limited in number and have not undergone evaluation before implementation. Indeed, a multilaboratory external quality assessment in late 2021 revealed gaps in the calling of contemporary variants that stemmed from an inadequate selection of diagnostic targets to discern between variants (27). Given this, highly multiplexed, efficient platforms are invaluable but are limited in number and warrant comprehensive evaluation before implementation in the molecular microbiology laboratory.

Here, we reported a comprehensive diagnostic evaluation of one of the highest multiplexed variant panel assays on the market. Based on a diverse cohort of clinical specimens across two continents and a wide timeline of the pandemic, we highlighted almost perfect levels of interrater agreement between this assay and the “gold standard” WGS for 9 of 11 variants and 25 of 30 distinct targets tested. The assay has a high diagnostic specificity across all variants (≥96.15%) and all targets (≥94.34%) tested. Furthermore, the panel showed high diagnostic specificity and sensitivity for contemporary variants in global circulation (e.g., Delta, Omicron [BA.1]).

Our study does present some limitations, particularly with respect to limited sampling. While the panel has defined target signatures for 16 different variants, we were only able to recover clinical specimens that corresponded to 11 of these variants for testing. Indeed, variants with the lowest level of agreement and diagnostic performance metrics were those with some of the fewest specimens recovered and tested (e.g., Zeta (*n* = 1), Beta (*n* = 4), Eta (*n* = 7)). We also did not include specimens from the early phase of the pandemic, including D614 viruses (33, 39), which limited diagnostic analyses of the D614G variant and individual targets. It is important to note, however, that the D614G polymorphism has undergone positive selection to eventuate emergent variants (40), and these older viruses have largely been replaced by the emergent Omicron lineage(s) (6, 41). We also recognize that we did not conduct this study at the extraction step of clinical specimens given the limited availability of remnant upper respiratory or saliva specimens.

A unique benefit of a highly multiplexed molecular assay is its adaptability to the natural evolution of the pathogen at hand, which confers the ability to identify changes in circulating viruses that manifest as distinct target result signatures. To assess this potential, we included undefined variants to determine if the discrete assay target result patterns could elucidate a variant’s identity without necessarily providing a defined result as the current software stands. Testing of Mu specimens resulted in a distinguishable combination of 5 detectable substitutions, but each result was interpreted as a Zeta (P.2) variant. This scenario highlights the utility of distinct target results to point to new viruses that rapidly arise in the circulating milieu of variants. However, this also underscores the need for adaptable target result interpretation software to address acute changes detected in patient populations. Furthermore, this flexibility is important to consider as variant classification methods are, themselves, inherently dynamic as they are shaped by the emergence, spread, and evolution of viral variants. We also tested clinical specimens that corresponded with the most current variant in circulation – the Omicron sublineage BA.2 – which has largely replaced BA.1 globally from January through April 2022 (https://covariants.org/, accessed April 26, 2022). From our results, we reported a BA.2-specific pattern of target results on this panel that could be used to readily discriminate the BA.2 from the BA.1 subtype. Indeed, future studies and the following months will be key to monitoring to understand the utility of this platform for capturing other emerging Omicron sublineages (e.g., BA.2.12.1, BA.3, BA.4, and BA.5).

Accurate identification of currently circulating and emerging SARS-CoV-2 variants is key to effective pathogen surveillance and providing optimal care to patients, but such methods must also be cost-effective to successfully implement. In our hands, after RNA extraction, we estimated the total cost of processing through variant calling by WGS to be $270 per specimen. This represents a per-specimen cost that is more than 10-fold more expensive than that estimated for the variant panel studied ($25). Although WGS is the mainstay for pathogen surveillance, this may not be a realistic technology for many LICs and LMCs, particularly in the context of a global pandemic (29). Therefore, rapid, cost-effective, conventional technologies (e.g., RT-PCR) are invaluable for the clinical laboratory. However, these also require increased diagnostic resolution to adequately capture viral evolution and meet the needs of pathogen surveillance. Thus, highly multiplexed molecular assays such as the one presented benefit from high discriminatory power and are a vital tool to shed light on changing viral dynamics.

## MATERIALS AND METHODS

### Ethics statement.

For specimens obtained through routine testing at MSHS, the Mount Sinai Pathogen Surveillance Program was reviewed and approved by the Human Research Protection Program at the Icahn School of Medicine at Mount Sinai (ISMMS) (HS number 13-00981). For specimens from Colombia, the study was reviewed and approved by the Ethics Committee from Universidad del Rosario in Bogotá, Colombia (Act number DVO005 1550-CV1499). This study was performed following the Declaration of Helsinki and its later amendments, and all patient data were anonymized to minimize the risk to participants.

### SARS-CoV-2 specimen collection and testing.

Residual viral RNA from a total of 391 specimens that were previously collected from September 2, 2020 to March 2, 2022 for routine diagnostic testing were utilized for this study.

Specifically, 349 upper respiratory tract (e.g., nasopharyngeal, anterior nares) and saliva (September 2, 2020 to March 2, 2022) specimens were originally collected for SARS-CoV-2 diagnostic testing in the Molecular Microbiology Laboratory of the MSHS Clinical Laboratory, which is certified under Clinical Laboratory Improvement Amendments of 1988, 42 U.S.C. §263a and meets requirements to perform high-complexity tests were eligible for inclusion in this study. Viral RNA was extracted from 300 μL of each specimen using the Viral DNA/RNA 300 kit H96 (PerkinElmer, CMG-1033-S) on the automated chemagic 360 instrument (PerkinElmer, 2024-0020) per the manufacturer’s protocol as previously described (42, 43). After routine testing and extraction, viral RNA was stored at −80°C before recovery for testing in this study.

Forty-two nasopharyngeal specimens were collected from patients from the Valle del Cauca department for SARS-CoV-2 testing at Universidad del Rosario from March 29, 2021 to July 28, 2021. Details of processing and SARS-CoV-2 testing of upper respiratory specimens have been described previously (44). After diagnostic testing, residual viral RNA was also stored at −80°C before recovery for testing in this study.

### SARS-CoV-2 sequencing, assembly, and phylogenetics.

As part of the ongoing Mount Sinai Pathogen Surveillance Program, SARS-CoV-2 viral RNA from MSHS underwent RT-PCR and next-generation sequencing followed by genome assembly and lineage assignment using a phylogenetic-based nomenclature as described by Rambaut et al. (45) using the Pangolin v4.0.6 tool and PANGO-v1.2.81 nomenclature scheme (https://github.com/cov-lineages/pangolin) as previously described (4, 46).

Sequence libraries were prepared from RNA from Colombian specimens using the ARTIC Network protocol (https://artic.network/ncov-2019, accessed February 1, 2021) as previously described (47). Briefly, long-read Oxford Nanopore MinION sequencing was conducted by the MinKNOW application (v1.5.5). Raw Fast5 files were base called and demultiplexed using Guppy. Reads were filtered to remove possible chimeric reads, and genome assemblies were obtained following the MinION pipeline described in the ARTIC bioinformatics pipeline (https://artic.network/ncov-2019/ncov2019-bioinformatics-sop.html, accessed February 1, 2021).

Of 391 specimens, 381 single variant consensus genome sequences were identified, and the remaining 10 yielded mixed assemblies. Thus, a putative (inconclusive) consensus genome sequence was generated. All FASTA consensus genome sequences underwent mutation calling and phylogenetic lineage assignment by the Nextclade Web Interface (https://clades.nextstrain.org/, accessed April 18, 2022) and the Pangolin COVID-19 Lineage Assigner (https://pangolin.cog-uk.io/, accessed April 18, 2022).

### SARS-CoV-2 variant panel testing.

We recovered residual viral RNA from all 391 specimens from −80°C storage to undergo testing on the Agena MassARRAY SARS-CoV-2 Variant Panel v3 (https://www.agenabio.com/products/panel/coronavirus-sars-cov-2-variant-detection-research-panel, accessed April 5, 2022). The panel combines RT-PCR and matrix-assisted laser desorption/ionization time-of-flight (MALDI-TOF) to detect targeted viral polymorphisms in the Spike (S) gene (Fig. 1A). It consists of a two-well multiplex qualitative assay that utilizes primer mixes that target a total of 36 polymorphisms, which reflected 16 distinct SARS-CoV-2 variants in various signature combinations (Fig. 1B). Briefly, after RNA extraction, this assay amplifies targeted regions in the viral genome by RT-PCR. As previously described (48), amplicons undergo a single nucleotide extension reaction using an extension primer (termed “probe” in the manuscript). Extension products are desalted and analyzed by MALDI-TOF mass spectrometry, which discriminates on specific masses of terminal nucleotides and overall extension products. Technical details of this process are described further below.

**(i) RT-PCR and generation of analytes.** Per the manufacturer’s protocol, for each specimen, viral RNA underwent RT-PCR by combining 0.355 μL nuclease-free water, 1 μL RT-PCR Mastermix, 0.125 μL RNase Inhibitor, and 0.020 μL of MMLV Enzyme in each of two wells in a 384-well format. To one well, 0.5 μL SARS-CoV-2 Variant v3 PCR Primer P01 was added. To the second well, 0.5 μL SARS-CoV-2 Variant v3 PCR Primer P02 was added. Three microliters of sample RNA were added to each of the two wells for a final RT-PCR volume of 5 μL. Four positive controls of synthetic SARS-CoV-2 RNA (Twist Synthetic SARS-CoV-2 RNA Controls 1 [MT007544.1, number 102019], 14 [B.1.1.7_710528, number 103907], 16 [EPI_ISL_678597, number 104043], and 17 [EPI_ISL_792683, number 104044]) were diluted in a mixture of nuclease-free water (Ambion number AM9916) and human liver total RNA (TaKaRa Bio number 636531) and included in each RT-PCR run. This resulted in a total of 8 wells with 1,500 SARS-CoV-2 genome copies/well and 10 ng human liver total RNA/well for all positive controls. Negative control of nuclease-free water was included in each RT-PCR run. RT-PCR thermocycler conditions are depicted in Table S1 in Supplemental File 1.

RT-PCR products underwent a reaction with shrimp alkaline phosphatase (SAP). Directly to each RT-PCR well, a mastermix of 1.53 μL nuclease-free water, 0.17 μL SAP Buffer, and 0.30 μL SAP was added for a total volume of 7 μL, including 2 μL of SAP mastermix. SAP reaction thermocycler conditions are described in Table S2 in Supplemental File 1.

Extension products were generated with SARS-CoV-2 Variant v3 Extend Primers using the iPLEX Pro Reagent Set. Mastermixes were created as follows: 1.06 μL nuclease-free water, 0.20 μL iPLEX Buffer Plus, GPR; 0.20 μL iPLEX Termination Mix, 0.04 μL iPLEX Pro Enzyme, and 0.50 μL MassARRAY SARS-CoV-2 Variant v3 Extend Primers (E01 or E02). Two microliters of E01 mastermix were added to each well amplified by P01 primers, and 2 μL E02 mastermix was added to each well amplified by P02 primers for the total extension reaction volume of 9 μL. Extension thermocycler conditions are detailed in Table S3 in Supplemental File 1.

**(ii) Analyte dispensing, data acquisition, and data analyses.** Twenty microliters of nuclease-free water were added to each well before desalting and dispensing in the MassARRAY System for data acquisition. Analytes were desalted using suspended clean resin (Agena number 08060) and dispensed onto SpectroCHIP Arrays (CPM-384) for data acquisition with the MassARRAY Analyzer with Chip Prep Module 384 as per the manufacturer’s protocol. Instrument settings for iPLEX Pro genotyping panels were used with Genotype+Area selected as the Process Method. After data acquisition, MassARRAY Typer Analyzer was used to analyze data and generate variant report output results for each specimen. All variant target results and individual target results for each specimen are depicted in Table S4 in Supplemental File 1.

### Diagnostic performance analyses.

To compare the performance of the panel to the WGS “gold standard,” we generated 2 × 2 contingency tables for detected and not detected results of each variant call or target call. To measure the level of agreement between WGS and the variant panel, we performed agreement analyses with kappa (κ) results and 95% confidence intervals (95% CI) using the publicly available GraphPad Prism web calculator (https://graphpad.com/quickcalcs/kappa2/, accessed April 20, 2022). The level of agreement was interpreted from kappa values as previously described (49). Interpretations included no (κ < 0), slight (0 ≤ κ ≤ 0.20), fair (0.21 ≤ κ ≤ 0.40), moderate (0.41 ≤ κ ≤ 0.60), substantial (0.61 ≤ κ ≤ 0.80), and almost perfect agreement (0.80 ≤ κ ≤ 1.00). In addition, we measured diagnostic sensitivity and specificity and negative (NPV) and positive predictive values (PPV) for each variant and individual target polymorphism tested (GraphPad Prism v.9.3.1). Ninety-five percent confidence intervals were calculated by the hybrid Wilson/Brown method. Fisher’s exact tests were performed for each contingency table for each variant and/or target tested.

Statistical analyses were performed for variant call results of 12 of the possible 16 variants on the panel. We also performed these analyses for 30 of the 36 possible targets on the panel as clinical specimens that encoded 6 specific amino acid polymorphisms (D80G, Y453F, E484Q, Q493K, N501T, and I692V) were not recovered for this study. In addition, we did not recover any specimens that harbored the native D614 amino acid (A23403 nucleotide), and, therefore, we were not able to compute the level of agreement or diagnostic specificity for the D614G variant or D614G target calls. In addition, for performance analyses of targets H69_V70del, N439K, and E484K, we excluded specimens that resulted in target dropout as we could not infer nucleotide polymorphisms that caused dropout given that primer/probe sequences are proprietary and not known.

To assess the prevalence of variant panel targets across Omicron sublineages, we interrogated publicly available SARS-CoV-2 genomes on the GISAID database (last accessed May 6, 2022). Using the online graphical user interface, we counted the number of genomes that harbored each of the 36 possible substitutions for each of four Omicron sublineages: BA.2.12.1 (*n* = 12324 genomes), BA.3 (*n* = 184), BA.4 (*n* = 857), BA.5 (*n* = 437). The prevalence of each substitution was determined by dividing the number of genomes with the substitution by the total number of genomes analyzed for the sublineage in question.

### Display items.

All figures are original and were generated using the GraphPad Prism software, Microsoft Excel v16.60, and finished in Adobe Illustrator (v.26.1). Fig. 1A was created in BioRender.com and finished in Adobe Illustrator.

### Data availability.

All single variant consensus genome sequences were deposited in the publicly available Global Initiative on Sharing Avian Influenza Data (GISAID) database (www.gisaid.org) (accession identifiers indicated in Table S4 in Supplemental File 1). The remaining 10 genomes with mixed patterns of mutations were not deposited into GISAID as single variant consensus genomes could not be resolved (data available upon request).
