# Supplementary material for: A Robust, Highly Multiplexed Mass Spectrometry Assay to Identify SARS-CoV-2 Variants
Source: Microbiol Spectr. 2022 Sep 7;10(5):e01736-22. doi: 10.1128/spectrum.01736-22 (PMC9604185; doi:10.1128/spectrum.01736-22)
Supplement: Supplemental file 1 — Tables S1 to S6. Download spectrum.01736-22-s0001.pdf, PDF file, 0.5 MB [file spectrum.01736-22-s0001.pdf]

SUPPLEMENTAL INFORMATION

Table S1. RT-PCR thermocycling conditions

Step	Description	Temperature	Time	Cycles
1	Uracil-DNA-glycosylase	25°C	5 minutes	1
2	RNA reverse-transcription	50°C	10 minutes	1
3	Polymerase activation	95°C	2 minutes	1
4	PCR	95°C	5 seconds	10
5		65°C (-1°C /cycle)	10 seconds	
6		72°C	5 seconds	
7		95°C	5 seconds	35
8		55°C	10 seconds	
9		72°C	5 seconds	
10	Final extension	72°C	5 minutes	1
11	Sample preservation	10°C	---	Hold

Table S2. SAP reaction conditions

Step	Description	Temperature	Time	Cycles
1	Dephosphorylation	37°C	10 minutes	1
2	Enzyme inactivation	85°C	5 minutes	1
3	Sample preservation	10°C	---	Hold

Table S3. Extension thermocycler conditions

Step	Description	Temperature	Time	Sub-Cycles	Cycles
1	Initial denaturation	95°C	30 seconds	1	
2	Denaturation	95°C	5 seconds	1	40
3	Annealing/Extension	52°C	5 seconds	5	
4	Denaturation	80°C	5 seconds		
5	Final extension	72°C	3 minutes	1	
6	Sample preservation	10°C	---	Hold	

Table S4. Agena MassARRAY® SARS-CoV-2 Variant Panel v3 (RUO) results

ID	Collection Date	GISAID Accession [a]	PANGO Lineage (WGS)	Agena Variant Results [b]	Genetic Markers Detected [c]
PV17741	9/2/20	EPI_ISL_802220	B.1.239	D614G Detected.	D614G | K417T | Q493K
PV17750	9/3/20	EPI_ISL_802222	B.1.1.434	D614G Detected.	D614G
PV17762	9/3/20	EPI_ISL_801862	B.1.1.50	D614G Detected.	D614G | K417T
PV17911	9/3/20	EPI_ISL_802223	B.1.1.1	D614G Detected.	D614G
PV17731	9/4/20	EPI_ISL_801922	B.1.240	D614G Detected.	D614G
PV17864	9/4/20	EPI_ISL_802225	B.1.1.432	D614G Detected.	D614G | H69_V70del_Dropout
PV17895	9/4/20	EPI_ISL_801893	B.1.2	D614G Detected.	D614G
PV17896	9/4/20	EPI_ISL_802226	B.1.2	D614G Detected.	D614G
PV17754	9/5/20	EPI_ISL_802228	B.1.2	D614G Detected.	D614G | D80G | L18F | N439K_Dropout | Q493K
PV17758	9/5/20	EPI_ISL_801905	B.1.1.265	D614G Detected.	D614G
PV17759	9/5/20	EPI_ISL_801881	B.1.240	D614G Detected.	D614G
PV17761	9/5/20	EPI_ISL_802229	B.1.2	D614G Detected.	D614G
PV19592	9/6/20	EPI_ISL_801941	B.1.1	D614G Detected.	D614G
PV18393	9/7/20	EPI_ISL_801926	B.1.1.434	D614G Detected.	D614G
PV17748	9/8/20	EPI_ISL_801874	B.1.369	D614G Detected.	D614G
PV17846	9/8/20	EPI_ISL_6491830	B.1	D614G Detected.	D614G
PV17879	9/8/20	EPI_ISL_802230	B.1.361	D614G Detected.	D614G
PV17897	9/8/20	EPI_ISL_802231	B.1	D614G Detected.	D614G
PV17921	9/8/20	EPI_ISL_802232	B.1.265	D614G Detected.	D614G | D80G | H69_V70del_Dropout | K417N | L18F
PV17923	9/8/20	EPI_ISL_802234	B.1.1.434	D614G Detected.	D614G
PV19595	9/8/20	EPI_ISL_802235	B.1.1	D614G Detected.	D614G
PV17905	9/9/20	EPI_ISL_802237	B.1.240	D614G Detected.	D614G
PV17924	9/9/20	EPI_ISL_801940	B.1.1	D614G Detected.	D614G
PV17881	9/10/20	EPI_ISL_801883	B.1.1.231	D614G Detected.	D614G
PV17882	9/10/20	EPI_ISL_802238	B.1.1.434	D614G Detected.	D614G

PV18993	9/10/20	EPI_ISL_802243	B.1	D614G Detected.	D614G
PV17858	9/11/20	EPI_ISL_801900	B.1.1	D614G Detected.	D614G
PV19366	9/28/20	EPI_ISL_801959	B.1.258	B.1.258 Detected. D614G Detected.	D614G | D80G | H69_V70del | L18F | N439K | Q493K
PV26936	1/25/21	EPI_ISL_1708896	P.2	Florida Detected.	E484K | K1191N | N439K_Dropout | Q493K
PV27007	1/26/21	EPI_ISL_1708926	B.1.351	Beta (B.1.351) Detected. D614G Detected.	A701V | D614G | D80A | E484K | K417N | L242_L244del | N501Y
PV27065	1/27/21	EPI_ISL_1708944	B.1.429	B.1.427/B.1.429 (20C.Cal) Detected. D614G Detected.	D614G | L452R | S13I | W152C
PV27047	1/28/21	EPI_ISL_1300758	B.1.526	D614G Detected. Iota (B.1.526) Detected.	A701V | D253G | D614G | E484K | T95I
PV27056	1/29/21	EPI_ISL_1300899	B.1.526	D614G Detected. Iota (B.1.526) Detected.	A701V | D253G | D614G | E484K | H69_V70del | L5F | T95I
PV27058	1/29/21	EPI_ISL_1300900	B.1.526	D614G Detected. Iota (B.1.526) Detected.	A701V | D253G | D614G | L5F | S477N | T95I
PV27103	1/29/21	EPI_ISL_1708950	B.1.2	Broad USA Detected. D614G Detected.	D614G | H69_V70del_Dropout | Q677P
PV27081	1/30/21	EPI_ISL_1301403	B.1.429	B.1.427/B.1.429 (20C.Cal) Detected. D614G Detected.	D614G | L452R | S13I | W152C
PV27085	1/30/21	EPI_ISL_1300902	B.1.526	D614G Detected. Iota (B.1.526) Detected.	A701V | D253G | D614G | L5F | T95I
PV27082	1/30/21	EPI_ISL_1301404	B.1.2	Broad USA Detected. D614G Detected.	D614G | E484-K/Q_Dropout | H69_V70del_Dropout | Q677P
PV27090	1/31/21	EPI_ISL_5253374	B.1.526	D614G Detected. Iota (B.1.526) Detected.	A701V | D253G | D614G | L5F | T95I
PV27094	1/31/21	EPI_ISL_1301407	B.1.429	B.1.427/B.1.429 (20C.Cal) Detected. D614G Detected.	D614G | L452R | W152C
PV27105	1/31/21	EPI_ISL_1708951	B.1.526	D614G Detected. Iota (B.1.526) Detected.	A701V | D253G | D614G | L5F | T95I
PV27109	2/2/21	EPI_ISL_1300804	B.1.1.7	Alpha (B.1.1.7) Detected. D614G Detected.	A570D | D614G | H69_V70del | N501Y | P681H | S982A | T716I | Y144del_Composite
PV27111	2/2/21	EPI_ISL_1300903	B.1.526	D614G Detected. Iota (B.1.526) Detected.	A701V | D253G | D614G | L5F | T95I
PV27533	2/2/21	EPI_ISL_1708957	B.1.1.7	Alpha (B.1.1.7) Detected. D614G Detected.	A570D | D614G | H69_V70del | N501Y | P681H | S982A | T716I | Y144del_Composite
PV27541	2/2/21	EPI_ISL_1708956	B.1.526	D614G Detected. Iota (B.1.526) Detected.	A701V | D253G | D614G | E484K | L5F | T95I
PV27526	2/3/21	EPI_ISL_1300904	B.1.526	D614G Detected. Iota (B.1.526) Detected.	A701V | D253G | D614G | L5F | T95I
PV27528	2/3/21	EPI_ISL_1300905	B.1.526	D614G Detected. Iota (B.1.526) Detected.	A701V | D253G | D614G | E484K | L5F | T95I
PV27535	2/3/21	EPI_ISL_1300907	B.1.526	D614G Detected. Iota (B.1.526) Detected.	A701V | D253G | D614G | L5F | T95I

PV27539	2/3/21	EPI_ISL_1300908	B.1.526	D614G Detected. Iota (B.1.526) Detected.	A701V | D253G | D614G | E484K | L5F | T95I
PV27534	2/3/21	EPI_ISL_1300906	B.1.526	D614G Detected. Iota (B.1.526) Detected.	A701V | D253G | D614G | E484K | L5F | T95I
PV27543	2/3/21	EPI_ISL_1300768	B.1.1.7	Alpha (B.1.1.7) Detected. D614G Detected.	A570D | D614G | H69_V70del | N501Y | P681H | S982A | T716I | Y144del_Composite
PV27547	2/4/21	EPI_ISL_1301418	B.1.427	B.1.427/B.1.429 (20C.Cal) Detected. D614G Detected.	D614G | L452R | S13I | W152C
PV27553	2/4/21	EPI_ISL_1300809	B.1.1.7	Alpha (B.1.1.7) Detected. D614G Detected.	A570D | D614G | H69_V70del | N501Y | P681H | S982A | T716I | Y144del_Composite
PV27549	2/4/21	EPI_ISL_1300909	B.1.526	D614G Detected. Iota (B.1.526) Detected.	A701V | D253G | D614G | E484K | L5F | T95I
PV27566	2/4/21	EPI_ISL_5253375	B.1.526	D614G Detected.	A701V | D614G | T95I
PV27567	2/4/21	EPI_ISL_5253376	B.1.526	D614G Detected. Iota (B.1.526) Detected.	A701V | D253G | D614G | L5F | T95I
PV27571	2/4/21	EPI_ISL_5336550	B.1.526	D614G Detected. Iota (B.1.526) Detected.	A701V | D253G | D614G | L5F | T95I
PV27573	2/4/21	EPI_ISL_1708964	B.1.1.7	Alpha (B.1.1.7) Detected. D614G Detected.	A570D | D614G | H69_V70del | N501Y | P681H | S982A | T716I | Y144del_Composite
PV27597	2/4/21	EPI_ISL_1708969	B.1.526	D614G Detected. Iota (B.1.526) Detected.	A701V | D253G | D614G | E484K | L5F | T95I
PV27587	2/5/21	EPI_ISL_1300910	B.1.526	D614G Detected. Iota (B.1.526) Detected.	A701V | D253G | D614G | E484K | L5F | T95I
PV27584	2/5/21	EPI_ISL_1301426	B.1.427	B.1.427/B.1.429 (20C.Cal) Detected. D614G Detected.	D614G | L452R | S13I | W152C
PV27589	2/5/21	EPI_ISL_1300912	B.1.526	D614G Detected. Iota (B.1.526) Detected.	A701V | D253G | D614G | E484K | L5F | T95I
PV27590	2/5/21	EPI_ISL_1300913	B.1.526	D614G Detected. Iota (B.1.526) Detected.	A701V | D253G | D614G | E484K | L5F | T95I
PV27593	2/5/21	EPI_ISL_1300914	B.1.526	D614G Detected. Iota (B.1.526) Detected.	A701V | D253G | D614G | E484K | L5F | T95I
PV27608	2/6/21	EPI_ISL_1300915	B.1.526	D614G Detected. Iota (B.1.526) Detected.	A701V | D253G | D614G | E484K | N439K_Dropout | T95I
PV27609	2/6/21	EPI_ISL_1300916	B.1.526	D614G Detected. Iota (B.1.526) Detected.	A701V | D253G | D614G | E484K | L5F | T95I
PV27610	2/6/21	EPI_ISL_1301429	B.1.429	B.1.427/B.1.429 (20C.Cal) Detected. D614G Detected.	D614G | L452R | S13I | W152C
PV27616	2/7/21	EPI_ISL_1300810	B.1.1.7	Alpha (B.1.1.7) Detected. D614G Detected. P.2 Detected.	A570D | D614G | E484K | H69_V70del | N501Y | P681H | S982A | T716I | Y144del_Composite
PV27618	2/7/21	EPI_ISL_1300818	B.1.526	D614G Detected. Iota (B.1.526) Detected.	A701V | D253G | D614G | E484K | L5F | T95I
PV27631	2/7/21	EPI_ISL_1300805	B.1.1.7	Alpha (B.1.1.7) Detected. D614G Detected.	A570D | D614G | H69_V70del | N501Y | P681H | S982A | T716I | Y144del_Composite

PV27651	2/7/21	EPI_ISL_1708981	B.1.526	D614G Detected. Iota (B.1.526) Detected. P.2 Detected.	A701V | D253G | D614G | E484K | L5F | S477N | T95I
PV27654	2/7/21	EPI_ISL_1708982	B.1.429	B.1.427/B.1.429 (20C.Cal) Detected. D614G Detected. P.2 Detected.	D614G | E484K | L452R | S13I | W152C
PV27671	2/7/21	EPI_ISL_1708984	B.1.526	D614G Detected. Iota (B.1.526) Detected.	A701V | D253G | D614G | E484K | L5F | T95I
PV27635	2/8/21	EPI_ISL_1301431	B.1.429	B.1.427/B.1.429 (20C.Cal) Detected. D614G Detected.	D614G | L452R | S13I | W152C
PV27643	2/8/21	EPI_ISL_1300917	B.1.526	D614G Detected. Iota (B.1.526) Detected.	A701V | D253G | D614G | E484K | L5F | T95I
PV27639	2/8/21	EPI_ISL_1300806	B.1.1.7	Alpha (B.1.1.7) Detected. D614G Detected.	A570D | A701V | D253G | D614G | H69_V70del | K1191N | N501Y | P681H | S982A | T716I | T95I | Y144del_Composite
PV27644	2/8/21	EPI_ISL_1300918	B.1.526	D614G Detected. Iota (B.1.526) Detected.	A701V | D253G | D614G | E484K | L5F | T95I
PV27645	2/8/21	EPI_ISL_1300919	B.1.526	D614G Detected. Iota (B.1.526) Detected.	A701V | D253G | D614G | E484K | L5F | T95I
PV27648	2/8/21	EPI_ISL_1300920	B.1.526	D614G Detected. Iota (B.1.526) Detected.	A701V | D253G | D614G | E484K | L5F | T95I
PV27646	2/8/21	EPI_ISL_1300762	B.1.526	D614G Detected. Iota (B.1.526) Detected.	A701V | D253G | D614G | E484K | L5F | T95I
PV27655	2/8/21	EPI_ISL_1708985	B.1.429	B.1.427/B.1.429 (20C.Cal) Detected. D614G Detected.	D614G | L452R | S13I | W152C
PV27653	2/9/21	EPI_ISL_1301432	B.1.429	B.1.427/B.1.429 (20C.Cal) Detected. B.1.526.1 Detected. D614G Detected.	D614G | D80G | L452R | S13I | W152C
PV27659	2/9/21	EPI_ISL_1300921	B.1.526	D614G Detected. Iota (B.1.526) Detected.	A701V | D253G | D614G | E484K | L5F | T95I
PV27666	2/9/21	EPI_ISL_1300811	B.1.1.7	Alpha (B.1.1.7) Detected. D614G Detected.	A570D | D614G | H69_V70del | N501Y | P681H | S982A | T716I | Y144del_Composite
PV27683	2/9/21	EPI_ISL_1708989	B.1.526	D614G Detected. Iota (B.1.526) Detected.	A701V | D253G | D614G | E484K | L5F | T95I
PV27684	2/9/21	EPI_ISL_1708991	B.1.1.7	Alpha (B.1.1.7) Detected. D614G Detected.	A570D | D614G | H69_V70del | N501Y | P681H | S982A | T716I | Y144del_Composite
PV27685	2/9/21	EPI_ISL_1708990	B.1.526	D614G Detected. Iota (B.1.526) Detected.	A701V | D253G | D614G | E484K | L5F | T95I
PV27675	2/10/21	EPI_ISL_5253420	B.1.526	D614G Detected. Iota (B.1.526) Detected.	A701V | D253G | D614G | E484K | L5F | T95I
PV27676	2/10/21	EPI_ISL_1300922	B.1.526	D614G Detected. Iota (B.1.526) Detected.	A701V | D253G | D614G | L5F | T95I
PV27682	2/10/21	EPI_ISL_1300923	B.1.526	D614G Detected. Iota (B.1.526) Detected.	A701V | D253G | D614G | E484K | L5F | T95I
PV27686	2/10/21	EPI_ISL_1300924	B.1.526	D614G Detected. Iota (B.1.526) Detected.	A701V | D253G | D614G | E484K | L5F | T95I

PV27680	2/10/21	EPI_ISL_1301438	B.1.429	B.1.427/B.1.429 (20C.Cal) Detected. D614G Detected.	D614G | L452R | S13I | W152C
PV27709	2/10/21	EPI_ISL_1708996	B.1.1.7	Alpha (B.1.1.7) Detected. D614G Detected.	A570D | D614G | H69_V70del | N501Y | P681H | S982A | T716I
PV27691	2/11/21	EPI_ISL_1300808	B.1.1.7	Alpha (B.1.1.7) Detected. D614G Detected.	A570D | D614G | H69_V70del | N501Y | P681H | S982A | T716I
PV27700	2/12/21	EPI_ISL_1300849	B.1.525	D614G Detected. Eta (B.1.525) Detected.	D614G | E484K | H69_V70del | Q677H | Y144del_Composite
PV27705	2/13/21	EPI_ISL_1300807	B.1.1.7	Alpha (B.1.1.7) Detected. D614G Detected.	A570D | D614G | H69_V70del | N501Y | P681H | S982A | T716I | Y144del_Composite
PV28428	2/16/21	EPI_ISL_1709006	B.1.429	B.1.427/B.1.429 (20C.Cal) Detected. D614G Detected.	D614G | L452R | S13I | W152C
PV28444	2/17/21	EPI_ISL_1709018	B.1.1.7	Alpha (B.1.1.7) Detected. D614G Detected.	A570D | D614G | H69_V70del | K1191N | N501Y | P681H | S982A | T716I | Y144del_Composite
PV28443	2/17/21	EPI_ISL_1709014	B.1.1.7	Alpha (B.1.1.7) Detected. D614G Detected.	A570D | D614G | H69_V70del | N501Y | P681H | S982A | T716I | Y144del_Composite
PV28450	2/17/21	EPI_ISL_1709021	B.1.1.7	Alpha (B.1.1.7) Detected. D614G Detected.	A570D | D614G | H69_V70del | N501Y | P681H | S982A | T716I | Y144del_Composite
PV28451	2/17/21	EPI_ISL_1709027	B.1.1.7	Alpha (B.1.1.7) Detected. D614G Detected.	A570D | D614G | H69_V70del | N501Y | P681H | S982A | T716I | Y144del_Composite
PV28459	2/17/21	EPI_ISL_1709029	B.1.1.7	Alpha (B.1.1.7) Detected. D614G Detected.	A570D | D614G | H69_V70del | L5F | N501Y | P681H | S982A | T716I | Y144del_Composite
PV28467	2/18/21	EPI_ISL_1709036	B.1.429	B.1.427/B.1.429 (20C.Cal) Detected. D614G Detected.	D614G | L452R | S13I | W152C
PV28480	2/19/21	EPI_ISL_1709043	B.1.1.7	Alpha (B.1.1.7) Detected. D614G Detected.	A570D | D614G | H69_V70del | N501Y | P681H | S982A | T716I | Y144del_Composite
PV28503	2/22/21	EPI_ISL_1709061	B.1.427	B.1.427/B.1.429 (20C.Cal) Detected. D614G Detected.	D614G | L452R | S13I | W152C
PV28500	2/22/21	EPI_ISL_1709060	B.1.1.7	Alpha (B.1.1.7) Detected. D614G Detected.	A570D | D614G | H69_V70del | N501Y | P681H | S982A | T716I | Y144del_Composite
PV28508	2/22/21	EPI_ISL_1709066	B.1.1.7	Alpha (B.1.1.7) Detected. D614G Detected.	A570D | A701V | D253G | D614G | H69_V70del | N501Y | P681H | S982A | T716I | T95I
PV28496	2/22/21	EPI_ISL_1709057	B.1.2	Broad USA Detected. D614G Detected.	D614G | Q677P
PV28507	2/23/21	EPI_ISL_5253379	B.1.1.7	Alpha (B.1.1.7) Detected. D614G Detected. P.2 Detected.	A570D | D614G | E484K | H69_V70del | L242_L244del | N501Y | P681H | S982A | T716I | Y144del_Composite

PV28864	3/2/21	EPI_ISL_1709159	B.1.2	Broad USA Detected. D614G Detected.	D614G | H69_V70del_Dropout | Q677P
PV35332	3/7/21	EPI_ISL_5253382	B.1.1.7	Alpha (B.1.1.7) Detected. D614G Detected. Iota (B.1.526) Detected. P.2 Detected.	A570D | A701V | D253G | D614G | E484K | H69_V70del | N501Y | P681H | T716I | T95I
PV35292	3/8/21	EPI_ISL_5253401	B.1.1.7	Alpha (B.1.1.7) Detected. D614G Detected.	A570D | D614G | H69_V70del | N501Y | P681H | S982A | T716I | Y144del_Composite
PV35293	3/8/21	EPI_ISL_1709255	C.37	D614G Detected.	D614G | T95I
PV35300	3/8/21	EPI_ISL_1709254	B.1.1.7	Alpha (B.1.1.7) Detected. D614G Detected.	A570D | D614G | H69_V70del | N501Y | P681H | S982A | T716I | Y144del_Composite
PV35339	3/8/21	EPI_ISL_5253419	B.1.429	B.1.427/B.1.429 (20C.Cal) Detected. D614G Detected.	D614G | L452R | S13I | W152C
PV35305	3/8/21	EPI_ISL_1709262	B.1.1.7	Alpha (B.1.1.7) Detected. D614G Detected.	A570D | D614G | H69_V70del | N501Y | P681H | S982A | T716I | Y144del_Composite
PV35319	3/9/21	EPI_ISL_5253381	B.1.1.7	Alpha (B.1.1.7) Detected. D614G Detected.	A570D | D614G | H69_V70del | N501Y | P681H | S982A | T716I | Y144del_Composite
PV35343	3/9/21	EPI_ISL_1709285	C.37	D614G Detected.	D614G
PV35324	3/10/21	EPI_ISL_1709286	C.37	D614G Detected.	D614G
PV35341	3/10/21	EPI_ISL_1709289	C.37	D614G Detected.	D614G
PV35149	3/10/21	EPI_ISL_1709296	C.37	D614G Detected.	D614G | T95I
PV35328	3/10/21	EPI_ISL_1709299	B.1.1.7	Alpha (B.1.1.7) Detected. D614G Detected.	A570D | D614G | H69_V70del | N501Y | P681H | S982A | T716I | Y144del_Composite
PV35346	3/10/21	EPI_ISL_1709297	B.1.1.7	Alpha (B.1.1.7) Detected. D614G Detected.	A570D | D614G | H69_V70del | N501Y | P681H | S982A | T716I | Y144del_Composite
PV35350	3/11/21	EPI_ISL_1709304	B.1.1.7	Alpha (B.1.1.7) Detected. D614G Detected.	A570D | D614G | H69_V70del | N501Y | P681H | S982A | T716I | Y144del_Composite
PV35552	3/19/21	EPI_ISL_1709529	B.1.2	Broad USA Detected. D614G Detected.	D614G | E484-K/Q_Dropout | H69_V70del_Dropout | Q677P
PV35915	3/21/21	EPI_ISL_1709556	C.37	D614G Detected.	D614G
PV35921	3/22/21	EPI_ISL_5253270	B.1.427	B.1.427/B.1.429 (20C.Cal) Detected. D614G Detected.	D614G | L452R | S13I | W152C
PV35926	3/22/21	EPI_ISL_1709570	B.1.1.7	Alpha (B.1.1.7) Detected. D614G Detected.	A570D | D614G | H69_V70del | N501Y | P681H | S982A | T716I
PV35928	3/22/21	EPI_ISL_1709572	B.1.1.7	Alpha (B.1.1.7) Detected. D614G Detected.	A570D | D614G | H69_V70del | N501Y | P681H | S982A | T716I | Y144del_Composite

PV35929	3/22/21	EPI_ISL_1709574	B.1.1.7	Alpha (B.1.1.7) Detected. D614G Detected.	A570D | D614G | H69_V70del | N501Y | P681H | S982A | T716I | Y144del_Composite
PV35931	3/22/21	EPI_ISL_1709577	B.1.525	D614G Detected.	D614G | E484K | H69_V70del_Dropout | Q677H | Y144del_Composite
PV35948	3/23/21	EPI_ISL_1709627	B.1.1.7	Alpha (B.1.1.7) Detected. D614G Detected.	A570D | D614G | H69_V70del | N501Y | P681H | S982A | T716I | Y144del_Composite
PV35953	3/23/21	EPI_ISL_1709625	P.1	D614G Detected. Gamma (P.1) Detected.	D614G | E484K | K417T | L18F | N501Y
PV35955	3/23/21	EPI_ISL_1709626	P.1	D614G Detected. Gamma (P.1) Detected.	D614G | E484K | K417T | L18F | N501Y
PV35947	3/24/21	EPI_ISL_5253383	B.1.1.7	Alpha (B.1.1.7) Detected. D614G Detected.	A570D | D614G | H69_V70del | N501Y | P681H | S982A | T716I | Y144del_Composite
PV35962	3/24/21	EPI_ISL_1709654	B.1.1.7	Alpha (B.1.1.7) Detected. D614G Detected.	A570D | D614G | H69_V70del | N501Y | P681H | S982A | T716I | Y144del_Composite
PV35963	3/24/21	EPI_ISL_1709652	B.1.1.7	Alpha (B.1.1.7) Detected. D614G Detected.	A570D | D614G | H69_V70del | N501Y | P681H | S982A | T716I | Y144del_Composite
PV35960	3/24/21	EPI_ISL_1709650	B.1.1.7	Alpha (B.1.1.7) Detected. D614G Detected.	A570D | D614G | H69_V70del | N501Y | P681H | S982A | T716I | Y144del_Composite
PV35957	3/25/21	EPI_ISL_5253378	B.1.1.7	Alpha (B.1.1.7) Detected. D614G Detected.	A570D | D614G | H69_V70del | L5F | N501Y | P681H | S982A | T716I | Y144del_Composite
PV35958	3/25/21	EPI_ISL_1709657	B.1.1.7	Alpha (B.1.1.7) Detected. D614G Detected.	A570D | D614G | H69_V70del | N501Y | P681H | S982A | T716I | Y144del_Composite
PV35970	3/25/21	EPI_ISL_5253272	B.1.429	B.1.427/B.1.429 (20C.Cal) Detected. D614G Detected.	D614G | L452R | S13I | W152C
PV35966	3/25/21	EPI_ISL_5253271	B.1.427	B.1.427/B.1.429 (20C.Cal) Detected. D614G Detected.	D614G | L452R | S13I | W152C
PV35977	3/25/21	EPI_ISL_1709686	B.1.427	B.1.427/B.1.429 (20C.Cal) Detected. D614G Detected.	D614G | L452R | S13I | W152C
A259	3/29/21	EPI_ISL_7307284	P.1	D614G Detected. Gamma (P.1) Detected.	D614G | E484K | K417T | L18F | N501Y
A260	3/29/21	EPI_ISL_7307290	P.1	D614G Detected. Gamma (P.1) Detected.	D614G | E484K | K417T | L18F | N501Y
PV36288	3/29/21	EPI_ISL_1709774	B.1.351	Beta (B.1.351) Detected. D614G Detected.	A701V | D614G | D80A | E484K | K417N | L18F | L242_L244del | N501Y
PV36213	3/30/21	EPI_ISL_1709785	B.1.351	Beta (B.1.351) Detected. Gamma (P.1) Detected.	A701V | D80A | E484K | K417N | K417T | L18F | N439K_Dropout | N501Y
PV36885	4/4/21	EPI_ISL_6492372	B.1.525	D614G Detected.	D614G | E484K | H69_V70del_Dropout | Q677H

PV31898	4/19/21	EPI_ISL_6492518	B.1.525	D614G Detected.	D614G | E484K | H69_V70del_Dropout | Q677H | Y144del_Composite
K12	4/23/21	EPI_ISL_7476873	C.37	D614G Detected.	D614G
K2	4/23/21	EPI_ISL_7476639	C.37	D614G Detected.	D614G
K3	4/23/21	EPI_ISL_7476266	C.37	D614G Detected.	D614G
K5	4/23/21	EPI_ISL_7476273	C.37	D614G Detected.	D614G
K6	4/23/21	EPI_ISL_7476401	C.37	D614G Detected.	D614G
K7	4/23/21	EPI_ISL_7476602	C.37	D614G Detected.	D614G
K18	4/26/21	EPI_ISL_7476877	C.37	D614G Detected.	D614G
K19	4/26/21	EPI_ISL_7476887	C.37	D614G Detected.	D614G
PV37801	4/29/21	EPI_ISL_6492619	B.1.525	D614G Detected.	D614G | E484K | H69_V70del_Dropout | Q677H
K13	5/1/21	EPI_ISL_7476805	C.37	D614G Detected.	D614G
K22	5/4/21	EPI_ISL_7476461	C.37	D614G Detected.	D614G | T95I
K23	5/4/21	EPI_ISL_7476811	C.37	D614G Detected.	D614G
K24	5/4/21	EPI_ISL_7476815	C.37	D614G Detected.	D614G
PV31950	5/5/21	EPI_ISL_6492667	B.1.525	D614G Detected.	D614G | E484K | H69_V70del_Dropout | Q677H | Y144del_Composite
PV31971	5/7/21	EPI_ISL_6492691	B.1.525	D614G Detected.	D614G | E484K | H69_V70del_Dropout | Q677H | Y144del_Composite
K25	5/10/21	EPI_ISL_7476823	C.37	D614G Detected.	D614G
K26	5/10/21	EPI_ISL_7476833	C.37	D614G Detected.	D614G
K27	5/10/21	EPI_ISL_7476836	C.37	D614G Detected.	D614G
H158	5/11/21	EPI_ISL_7307276	P.1	D614G Detected. Gamma (P.1) Detected.	D614G | E484K | K417T | L18F | N501Y
K43	5/18/21	EPI_ISL_7476423	B.1.621	D614G Detected. P.2 Detected.	D614G | E484K | N501Y | P681H | T95I | Y144del_Composite
K47	5/18/21	EPI_ISL_7476562	B.1.621	D614G Detected. P.2 Detected.	D614G | E484K | N501Y | P681H | T95I | Y144del_Composite
K42	5/21/21	EPI_ISL_7476627	B.1.621	D614G Detected. P.2 Detected.	D614G | E484K | N501Y | P681H | Q677H | T95I | Y144del_Composite
K45	5/21/21	EPI_ISL_7476326	B.1.621	D614G Detected. P.2 Detected.	D614G | E484K | N501Y | P681H | T95I | Y144del_Composite

K46	5/21/21	EPI_ISL_7476345	B.1.621	D614G Detected. P.2 Detected.	D614G | E484K | N501Y | P681H | Q677H | T95I | Y144del_Composite
K49	5/29/21	EPI_ISL_7476302	B.1.621	D614G Detected. P.2 Detected.	D614G | E484K | N501Y | P681H | T95I | Y144del_Composite
K50	5/29/21	EPI_ISL_7476680	B.1.621	D614G Detected. P.2 Detected.	D614G | E484K | N501Y | P681H | Q677H | T95I | Y144del_Composite
K53	5/29/21	EPI_ISL_7476285	B.1.621	D614G Detected. P.2 Detected.	D614G | E484K | N501Y | P681H | T95I | Y144del_Composite
K54	5/29/21	EPI_ISL_7476482	B.1.621	D614G Detected. P.2 Detected.	D614G | E484K | N501Y | P681H | T95I | Y144del_Composite
K56	5/29/21	EPI_ISL_7476796	B.1.621	D614G Detected. P.2 Detected.	D614G | E484K | N501Y | P681H | T95I | Y144del_Composite
K57	5/29/21	EPI_ISL_7476365	B.1.621	D614G Detected. P.2 Detected.	D614G | E484K | N501Y | P681H | T95I | Y144del_Composite
K68	6/2/21	EPI_ISL_7476635	P.1	D614G Detected. Gamma (P.1) Detected.	D614G | E484K | K417T | L18F | N501Y
K59	6/4/21	EPI_ISL_7476556	P.1	D614G Detected. Gamma (P.1) Detected.	D614G | E484K | K417T | L18F | N501Y
K61	6/4/21	EPI_ISL_7476340	B.1.621	D614G Detected. P.2 Detected.	D614G | E484K | N501Y | P681H | T95I | Y144del_Composite
K62	6/4/21	EPI_ISL_7476181	B.1.621	D614G Detected. P.2 Detected.	D614G | E484K | N501Y | P681H | T95I | Y144del_Composite
K63	6/4/21	EPI_ISL_7476357	B.1.621	D614G Detected. P.2 Detected.	D614G | E484K | N501Y | P681H | T95I | Y144del_Composite
H100	6/16/21	EPI_ISL_7307242	P.1	D614G Detected. Gamma (P.1) Detected.	D614G | E484K | K417T | L18F | N501Y
H99	6/16/21	EPI_ISL_7307289	P.1	D614G Detected. Gamma (P.1) Detected.	D614G | E484K | K417T | L18F | N501Y
K95	6/21/21	EPI_ISL_7476174	P.1	D614G Detected. Gamma (P.1) Detected.	D614G | E484K | K417T | L18F | N501Y
K92	6/23/21	EPI_ISL_7476661	P.1	D614G Detected. Gamma (P.1) Detected.	D614G | E484K | K417T | L18F | N501Y
H119	7/4/21	EPI_ISL_7307241	P.1	D614G Detected. Gamma (P.1) Detected.	D614G | E484K | K417T | L18F | N501Y
K115	7/6/21	EPI_ISL_7476570	P.1	D614G Detected. Gamma (P.1) Detected.	D614G | E484K | K417T | L18F | N501Y
V17	7/22/21	EPI_ISL_12084490	P.1	D614G Detected. Gamma (P.1) Detected.	D614G | E484K | K417T | L18F | N501Y
K38	7/28/21	EPI_ISL_7476253	B.1.621	D614G Detected. P.2 Detected.	D614G | E484K | N501Y | P681H | T95I | Y144del_Composite
PV40356	11/1/21	EPI_ISL_6494430	AY.44	D614G Detected. Delta (B.1.617.2) Detected.	D614G | L452R | P681R | T19R | T478K

PV40371	11/1/21	EPI_ISL_6494434	AY.103	D614G Detected. Delta (B.1.617.2) Detected.	D614G | L452R | P681R | T19R | T478K
PV40360	11/1/21	EPI_ISL_6494432	AY.103	D614G Detected. Delta (B.1.617.2) Detected.	D614G | L452R | P681R | T19R | T478K
PV40358	11/1/21	EPI_ISL_6494431	AY.118	D614G Detected. Delta (B.1.617.2) Detected.	D614G | L452R | P681R | T19R | T478K | T95I
PV40364	11/1/21	EPI_ISL_6494433	AY.25	D614G Detected. Delta (B.1.617.2) Detected.	D614G | L452R | P681R | T19R | T478K
PV40351	11/2/21	EPI_ISL_12131519	B.1.617.2	D614G Detected. Delta (B.1.617.2) Detected.	D614G | L452R | P681R | T19R | T478K
PV40365	11/2/21	EPI_ISL_6494439	AY.103	D614G Detected. Delta (B.1.617.2) Detected.	D614G | L452R | P681R | T19R | T478K
PV40368	11/2/21	EPI_ISL_6494440	AY.103	D614G Detected. Delta (B.1.617.2) Detected.	D614G | L452R | P681R | T19R | T478K | T95I
PV40344	11/2/21	EPI_ISL_6494437	AY.100	D614G Detected. Delta (B.1.617.2) Detected.	D614G | L452R | P681R | T19R | T478K | T95I
PV40345	11/2/21	EPI_ISL_6494438	AY.103	D614G Detected. Delta (B.1.617.2) Detected.	D614G | L452R | P681R | T19R | T478K
PV40331	11/2/21	EPI_ISL_6494436	AY.100	D614G Detected. Delta (B.1.617.2) Detected.	D614G | L452R | P681R | T19R | T478K | T95I
PV40333	11/3/21	Mixed	AY.103	D614G Detected. Delta (B.1.617.2) Detected.	D614G | L452R | P681R | T19R | T478K
PV40337	11/3/21	Mixed	AY.103	D614G Detected. Delta (B.1.617.2) Detected.	D614G | L452R | P681R | T19R | T478K | T95I
PV40330	11/3/21	Mixed	B.1.617.2	D614G Detected. Delta (B.1.617.2) Detected.	D614G | L452R | P681R | T19R | T478K | T95I
PV40328	11/3/21	EPI_ISL_6494447	AY.44	D614G Detected. Delta (B.1.617.2) Detected.	D614G | L452R | P681R | T19R | T478K
PV40370	11/4/21	EPI_ISL_6494450	AY.103	D614G Detected. Delta (B.1.617.2) Detected.	D614G | L452R | P681R | T19R | T478K
PV40336	11/4/21	Mixed	AY.121	D614G Detected. Delta (B.1.617.2) Detected.	D614G | L452R | P681R | T19R | T478K | T95I
PV40334	11/4/21	EPI_ISL_6494449	AY.121	D614G Detected. Delta (B.1.617.2) Detected.	D614G | L452R | P681R | T19R | T478K | T95I
PV40329	11/4/21	EPI_ISL_6494448	AY.103	D614G Detected. Delta (B.1.617.2) Detected.	D614G | L452R | P681R | T19R | T478K
PV40327	11/4/21	EPI_ISL_12131518	AY.3	D614G Detected. Delta (B.1.617.2) Detected.	D614G | L452R | P681R | T19R | T478K
PV40338	11/5/21	EPI_ISL_6494454	AY.119	D614G Detected. Delta (B.1.617.2) Detected.	D614G | L452R | P681R | T19R | T478K
PV40317	11/5/21	EPI_ISL_6494451	B.1.617.2	D614G Detected. Delta (B.1.617.2) Detected.	D614G | L452R | P681R | T19R | T478K | T95I
PV40325	11/5/21	EPI_ISL_6494453	AY.117	D614G Detected.	D614G | E484-K/Q_Dropout | H69_V70del_Dropout | L452R | T478K | T95I
PV40341	11/5/21	EPI_ISL_6494455	AY.33	D614G Detected. Delta (B.1.617.2) Detected.	D614G | L452R | P681R | T19R | T478K
PV40319	11/5/21	EPI_ISL_6494452	AY.33	D614G Detected. Delta (B.1.617.2) Detected.	D614G | L452R | P681R | T19R | T478K
PV40323	11/5/21	EPI_ISL_12131516	AY.44	D614G Detected. Delta (B.1.617.2) Detected.	D614G | L452R | P681R | T19R | T478K
PV41850	11/6/21	EPI_ISL_7907853	AY.103	D614G Detected. Delta (B.1.617.2) Detected.	D614G | L452R | T19R | T478K

PV41851	11/6/21	EPI_ISL_7907956	AY.122	D614G Detected. Delta (B.1.617.2) Detected.	D614G | L452R | P681R | T19R | T478K | T95I
PV40315	11/6/21	EPI_ISL_6494458	AY.39	D614G Detected. Delta (B.1.617.2) Detected.	D614G | L452R | P681R | T19R | T478K | T95I
PV40324	11/7/21	EPI_ISL_12131517	AY.103	D614G Detected. Delta (B.1.617.2) Detected.	D614G | L452R | P681R | T19R | T478K
PV40318	11/7/21	EPI_ISL_12131515	AY.103	D614G Detected. Delta (B.1.617.2) Detected.	D614G | L452R | P681R | T19R | T478K
PV41854	11/7/21	EPI_ISL_7907957	AY.103	D614G Detected. Delta (B.1.617.2) Detected.	D614G | L452R | N439K_Dropout | P681R | T19R | T478K | T95I
PV41853	11/8/21	EPI_ISL_7907923	AY.119.2	D614G Detected. Delta (B.1.617.2) Detected.	D614G | L452R | P681R | T19R | T478K | T95I
PV41857	11/8/21	EPI_ISL_7907902	AY.3	D614G Detected. Delta (B.1.617.2) Detected.	D614G | L452R | P681R | T19R | T478K
PV41860	11/8/21	EPI_ISL_7907852	AY.103	D614G Detected. Delta (B.1.617.2) Detected.	D614G | L452R | P681R | T19R | T478K
PV41870	11/9/21	EPI_ISL_7907960	AY.47	D614G Detected. Delta (B.1.617.2) Detected.	D614G | L452R | P681R | T19R | T478K
PV41889	11/9/21	Mixed	AY.43	D614G Detected. Delta (B.1.617.2) Detected.	D614G | L452R | P681R | T19R | T478K
PV41900	11/9/21	EPI_ISL_7907929	AY.109	D614G Detected. Delta (B.1.617.2) Detected.	D614G | L452R | P681R | T19R | T478K | T95I
PV41868	11/9/21	EPI_ISL_7907958	AY.20	D614G Detected. Delta (B.1.617.2) Detected.	D614G | L452R | P681R | T19R | T478K | T95I
PV41881	11/9/21	EPI_ISL_7907892	AY.25	D614G Detected. Delta (B.1.617.2) Detected.	D614G | L452R | L5F | P681R | T19R | T478K
PV41871	11/9/21	EPI_ISL_7907914	AY.25.1	D614G Detected. Delta (B.1.617.2) Detected.	D614G | L452R | P681R | T19R | T478K
PV41903	11/10/21	EPI_ISL_7907965	AY.26	D614G Detected. Delta (B.1.617.2) Detected.	D614G | L452R | P681R | T19R | T478K
PV41904	11/10/21	EPI_ISL_7907854	AY.26	D614G Detected. Delta (B.1.617.2) Detected.	D614G | L452R | P681R | T19R | T478K
PV41872	11/10/21	EPI_ISL_7907883	AY.47	D614G Detected. Delta (B.1.617.2) Detected.	D614G | L452R | P681R | T19R | T478K
PV41878	11/10/21	EPI_ISL_7907884	AY.47	D614G Detected. Delta (B.1.617.2) Detected.	D614G | L452R | P681R | T19R | T478K
PV41869	11/10/21	EPI_ISL_7907882	AY.47	D614G Detected.	D614G | E484-K/Q_Dropout | H69_V70del_Dropout | L452R | T478K
PV41895	11/10/21	EPI_ISL_7907926	AY.118	D614G Detected. Delta (B.1.617.2) Detected.	D614G | L452R | P681R | T19R | T478K | T95I
PV41874	11/10/21	EPI_ISL_7907924	AY.103	D614G Detected. Delta (B.1.617.2) Detected.	D614G | L452R | P681R | T19R | T478K
PV41898	11/10/21	EPI_ISL_7907927	AY.103	D614G Detected. Delta (B.1.617.2) Detected.	D614G | L452R | P681R | T19R | T478K
PV41896	11/10/21	EPI_ISL_7908115	AY.4	D614G Detected. Delta (B.1.617.2) Detected.	D614G | L452R | P681R | T19R | T478K | T95I
PV41862	11/10/21	EPI_ISL_7907988	AY.44	D614G Detected. Delta (B.1.617.2) Detected.	D614G | H69_V70del | L452R | P681R | T19R | T478K
PV41861	11/10/21	EPI_ISL_7907982	AY.39	D614G Detected. Delta (B.1.617.2) Detected.	D614G | L452R | P681R | T19R | T478K | T95I
PV41876	11/10/21	EPI_ISL_7907915	AY.103	D614G Detected. Delta (B.1.617.2) Detected.	D614G | L452R | P681R | T19R | T478K

PV41899	11/11/21	EPI_ISL_7907928	AY.100	D614G Detected. Delta (B.1.617.2) Detected.	D614G | L452R | P681R | T19R | T478K | T95I
PV41928	11/11/21	EPI_ISL_7907886	AY.103	D614G Detected. Delta (B.1.617.2) Detected.	D614G | L452R | P681R | T19R | T478K
PV41887	11/11/21	EPI_ISL_7907962	AY.44	D614G Detected. Delta (B.1.617.2) Detected.	D614G | L452R | P681R | T19R | T478K
PV41893	11/11/21	EPI_ISL_7907963	AY.103	D614G Detected. Delta (B.1.617.2) Detected.	D614G | L452R | P681R | T19R | T478K | T95I
PV41918	11/11/21	EPI_ISL_7907966	AY.25.1	D614G Detected. Delta (B.1.617.2) Detected.	D614G | L452R | P681R | T19R | T478K
PV41912	11/11/21	EPI_ISL_7907931	AY.3.3	D614G Detected. Delta (B.1.617.2) Detected.	D614G | L452R | P681R | T19R | T478K | T95I
PV41886	11/11/21	EPI_ISL_7907961	AY.3	D614G Detected. Delta (B.1.617.2) Detected.	D614G | L452R | P681R | T19R | T478K
PV41902	11/11/21	EPI_ISL_7907964	AY.3	D614G Detected.	D614G | E484-K/Q_Dropout | H69_V70del_Dropout | L452R | T478K
PV41873	11/11/21	EPI_ISL_7908114	B.1.351	D614G Detected.	A701V | D614G | E484K | H69_V70del_Dropout | N439K_Dropout | N501Y
PV41925	11/12/21	EPI_ISL_7907903	AY.9.2	D614G Detected. Delta (B.1.617.2) Detected.	D614G | L452R | P681R | T19R | T478K
PV41890	11/12/21	EPI_ISL_7907925	AY.126	D614G Detected. Delta (B.1.617.2) Detected.	D614G | L452R | P681R | T19R | T478K | T95I
PV41908	11/12/21	EPI_ISL_7907916	AY.103	D614G Detected. Delta (B.1.617.2) Detected.	D614G | L452R | P681R | T19R | T478K | T95I
PV41917	11/12/21	EPI_ISL_7907917	AY.103	D614G Detected. Delta (B.1.617.2) Detected.	D614G | L452R | P681R | T19R | T478K
PV41923	11/12/21	Mixed	AY.103	D614G Detected. Delta (B.1.617.2) Detected.	D614G | L452R | P681R | T19R | T478K
PV41926	11/12/21	EPI_ISL_7907967	AY.3	D614G Detected. Delta (B.1.617.2) Detected.	D614G | L452R | P681R | T19R | T478K
PV41929	11/13/21	EPI_ISL_7907932	AY.103	D614G Detected. Delta (B.1.617.2) Detected.	D614G | L452R | P681R | T19R | T478K
PV42315	11/13/21	EPI_ISL_7907969	AY.103	D614G Detected. Delta (B.1.617.2) Detected.	D614G | L452R | P681R | T19R | T478K
PV42312	11/13/21	EPI_ISL_7907936	AY.103	D614G Detected. Delta (B.1.617.2) Detected.	D614G | L452R | P681R | T19R | T478K
PV42295	11/14/21	EPI_ISL_7907887	AY.103	D614G Detected. Delta (B.1.617.2) Detected.	D614G | L452R | P681R | T19R | T478K
PV42311	11/14/21	EPI_ISL_7907893	AY.44	D614G Detected. Delta (B.1.617.2) Detected.	D614G | L452R | P681R | T19R | T478K
PV42313	11/15/21	EPI_ISL_7907968	AY.119	D614G Detected.	D614G | E484-K/Q_Dropout | H69_V70del_Dropout | L452R | T478K | T95I
PV42299	11/15/21	EPI_ISL_7907904	AY.25	D614G Detected. Delta (B.1.617.2) Detected.	D614G | L452R | P681R | T19R | T478K
PV42300	11/15/21	EPI_ISL_7907905	AY.106	D614G Detected. Delta (B.1.617.2) Detected.	D614G | L452R | L5F | P681R | T19R | T478K
PV42317	11/16/21	EPI_ISL_7907937	AY.44	D614G Detected. Delta (B.1.617.2) Detected.	D614G | L452R | P681R | T19R | T478K
PV42341	11/16/21	EPI_ISL_7907895	AY.122	D614G Detected. Delta (B.1.617.2) Detected.	D614G | L452R | P681R | T19R | T478K

PV42351	11/17/21	Mixed	AY.75	D614G Detected. Delta (B.1.617.2) Detected.	D614G | L452R | N439K_Dropout | P681R | T19R | T478K
PV42345	11/17/21	Mixed	AY.39	D614G Detected. Delta (B.1.617.2) Detected.	D614G | L452R | P681R | T19R | T478K | T95I
PV42355	11/17/21	EPI_ISL_7907920	AY.25	D614G Detected. Delta (B.1.617.2) Detected.	D614G | L452R | P681R | T19R | T478K
PV42348	11/17/21	EPI_ISL_7907888	AY.43	D614G Detected. Delta (B.1.617.2) Detected.	D614G | L452R | P681R | T19R | T478K | T95I
PV42346	11/17/21	EPI_ISL_7907987	AY.44	D614G Detected. Delta (B.1.617.2) Detected.	D614G | L452R | P681R | T19R | T478K
PV42340	11/17/21	EPI_ISL_7907940	AY.118	D614G Detected. Delta (B.1.617.2) Detected.	D614G | L452R | P681R | T19R | T478K | T95I
PV42335	11/17/21	EPI_ISL_7907919	AY.25	D614G Detected. Delta (B.1.617.2) Detected.	D614G | L452R | P681R | T19R | T478K
PV42336	11/17/21	EPI_ISL_7907970	AY.3.1	D614G Detected. Delta (B.1.617.2) Detected.	D614G | L452R | P681R | T19R | T478K
PV42338	11/17/21	EPI_ISL_7907894	AY.3.1	D614G Detected. Delta (B.1.617.2) Detected.	D614G | L452R | P681R | T19R | T478K
PV42356	11/17/21	Mixed	AY.43	D614G Detected. Delta (B.1.617.2) Detected.	D614G | L452R | P681R | T19R | T478K
PV42337	11/17/21	EPI_ISL_7907939	AY.122	D614G Detected. Delta (B.1.617.2) Detected.	D614G | L452R | P681R | T19R | T478K
PV42360	11/18/21	EPI_ISL_7908121	AY.103	D614G Detected. Delta (B.1.617.2) Detected.	D614G | L452R | P681R | T19R | T478K
PV42362	11/18/21	EPI_ISL_7908129	AY.103	D614G Detected. Delta (B.1.617.2) Detected.	D614G | L452R | P681R | T19R | T478K
PV42364	11/18/21	EPI_ISL_7907971	AY.100	D614G Detected. Delta (B.1.617.2) Detected.	D614G | N439K_Dropout | P681R | T19R | T478K | T95I
PV42366	11/18/21	EPI_ISL_7908131	AY.103	D614G Detected. Delta (B.1.617.2) Detected.	D614G | L452R | P681R | T19R | T478K
PV42378	11/19/21	EPI_ISL_7907941	AY.103	D614G Detected. Delta (B.1.617.2) Detected.	D614G | L452R | T19R | T478K
PV42382	11/19/21	EPI_ISL_7908120	AY.3	D614G Detected. Delta (B.1.617.2) Detected.	D614G | L452R | P681R | T19R | T478K
PV42375	11/19/21	EPI_ISL_7907972	AY.3	D614G Detected. Delta (B.1.617.2) Detected.	D614G | L452R | P681R | T19R | T478K
PV42377	11/19/21	EPI_ISL_7907889	AY.25.1	D614G Detected. Delta (B.1.617.2) Detected.	D614G | L452R | P681R | T19R | T478K
PV45513	12/13/21	EPI_ISL_7908098	BA.1	D614G Detected. Omicron (B.1.1.529) Detected.	D614G | H69_V70del_Dropout | K417N | N439K_Dropout | P681H | S477N | T478K | T95I | Y144del_Composite
PV45522	12/13/21	EPI_ISL_7908067	BA.1	D614G Detected. Omicron (B.1.1.529) Detected.	D614G | H69_V70del_Dropout | K417N | N439K_Dropout | P681H | S477N | T478K | T95I | Y144del_Composite
PV45515	12/13/21	EPI_ISL_7908066	BA.1	D614G Detected. Omicron (B.1.1.529) Detected.	D614G | H69_V70del_Dropout | K417N | N439K_Dropout | P681H | S477N | T478K | T95I | Y144del_Composite
PV45462	12/13/21	EPI_ISL_7908017	BA.1	D614G Detected. Omicron (B.1.1.529) Detected.	D614G | H69_V70del_Dropout | K417N | N439K_Dropout |

					P681H | S477N | T478K | T95I | Y144del_Composite
PV45475	12/14/21	EPI_ISL_7907891	AY.25.1	D614G Detected. Delta (B.1.617.2) Detected.	D614G | L452R | P681R | T19R | T478K
PV45464	12/14/21	EPI_ISL_7907980	AY.100	D614G Detected. Delta (B.1.617.2) Detected.	D614G | L452R | P681R | T19R | T478K | T95I
PV45451	12/14/21	EPI_ISL_7907986	AY.44	D614G Detected. Delta (B.1.617.2) Detected.	D614G | E484-K/Q_Dropout | L452R | P681R | T19R
PV45468	12/14/21	EPI_ISL_7907954	AY.43	D614G Detected. Delta (B.1.617.2) Detected.	D614G | L452R | P681R | T19R | T478K
PV45473	12/14/21	EPI_ISL_7907955	AY.3	D614G Detected. Delta (B.1.617.2) Detected.	D614G | L452R | P681R | T19R | T478K
PV45430	12/14/21	EPI_ISL_7907952	AY.43	D614G Detected. Delta (B.1.617.2) Detected.	D614G | L452R | P681R | T19R | T478K
PV45503	12/14/21	EPI_ISL_7908060	AY.3	D614G Detected. Delta (B.1.617.2) Detected.	D614G | L452R | P681R | T19R | T478K
PV45448	12/14/21	EPI_ISL_7907953	AY.103	D614G Detected. Delta (B.1.617.2) Detected.	D614G | L452R | P681R | T19R | T478K
PV45453	12/14/21	EPI_ISL_7908003	BA.1.1	D614G Detected. Omicron (B.1.1.529) Detected.	D614G | H69_V70del_Dropout | K417N | N439K_Dropout | P681H | S477N | T478K | T95I | Y144del_Composite
PV45497	12/14/21	EPI_ISL_7908074	BA.1	D614G Detected. Omicron (B.1.1.529) Detected.	D614G | H69_V70del_Dropout | K417N | N439K_Dropout | P681H | S477N | T478K | T95I | Y144del_Composite
PV45477	12/14/21	EPI_ISL_7908090	BA.1	D614G Detected. Omicron (B.1.1.529) Detected.	D614G | H69_V70del_Dropout | K417N | N439K_Dropout | P681H | S477N | T478K | T95I | Y144del_Composite
PV45466	12/14/21	EPI_ISL_7908088	BA.1.1	D614G Detected. Omicron (B.1.1.529) Detected.	D614G | H69_V70del_Dropout | K417N | N439K_Dropout | P681H | S477N | T478K | T95I | Y144del_Composite
PV45454	12/14/21	EPI_ISL_11178969	BA.1.1	D614G Detected. Delta (AY.1/AY.2) Detected.	D614G | K417N | P681H | S477N | T19R | T478K | T95I
PV45460	12/14/21	EPI_ISL_7908004	BA.1	D614G Detected. Omicron (B.1.1.529) Detected.	D614G | H69_V70del_Dropout | K417N | N439K_Dropout | P681H | S477N | T478K | T95I | Y144del_Composite
PV45452	12/14/21	EPI_ISL_7908043	BA.1	D614G Detected. Omicron (B.1.1.529) Detected.	D614G | H69_V70del_Dropout | K417N | N439K_Dropout | P681H | S477N | T478K | T95I | Y144del_Composite
PV45446	12/14/21	EPI_ISL_7908065	BA.1	D614G Detected. Omicron (B.1.1.529) Detected.	D614G | H69_V70del_Dropout | K417N | N439K_Dropout | P681H | S477N | T478K | T95I | Y144del_Composite
PV45489	12/14/21	EPI_ISL_7908096	BA.1.1	D614G Detected. Omicron (B.1.1.529) Detected.	D614G | H69_V70del_Dropout | K417N | N439K_Dropout | P681H | S477N | T478K | T95I | Y144del_Composite

PV45467	12/14/21	EPI_ISL_7908045	BA.1	D614G Detected. Omicron (B.1.1.529) Detected.	D614G | H69_V70del | K417N | N439K_Dropout | P681H | S477N | T478K | T95I | Y144del_Composite
PV45459	12/14/21	EPI_ISL_7908070	BA.1	D614G Detected. Omicron (B.1.1.529) Detected.	D614G | H69_V70del_Dropout | K417N | N439K_Dropout | P681H | S477N | T478K | T95I | Y144del_Composite
PV45471	12/14/21	EPI_ISL_7908089	BA.1	D614G Detected.	D614G | E484-K/Q_Dropout | H69_V70del_Dropout | N439K_Dropout | S477N | T478K | T95I
PV45472	12/14/21	EPI_ISL_7908071	BA.1	D614G Detected. Omicron (B.1.1.529) Detected.	A701V | D614G | H69_V70del | K417N | N439K_Dropout | P681H | S477N | T478K | T95I | Y144del_Composite
PV45458	12/14/21	EPI_ISL_7908086	BA.1.1	D614G Detected. Omicron (B.1.1.529) Detected.	D614G | H69_V70del_Dropout | K417N | N439K_Dropout | P681H | S477N | T478K | T95I | Y144del_Composite
PV45465	12/14/21	EPI_ISL_7908087	BA.1	D614G Detected. Omicron (B.1.1.529) Detected.	D614G | H69_V70del_Dropout | K417N | N439K_Dropout | P681H | S477N | T478K | T95I | Y144del_Composite
PV45478	12/14/21	EPI_ISL_7908091	BA.1	D614G Detected. Omicron (B.1.1.529) Detected.	D614G | H69_V70del_Dropout | K417N | N439K_Dropout | P681H | S477N | T478K | T95I | Y144del_Composite
PV45506	12/14/21	EPI_ISL_7908109	BA.1	D614G Detected. Omicron (B.1.1.529) Detected.	D614G | H69_V70del_Dropout | K417N | N439K_Dropout | P681H | S477N | T478K | T95I | Y144del_Composite
PV45490	12/14/21	EPI_ISL_7908097	BA.1	D614G Detected. Omicron (B.1.1.529) Detected.	D614G | H69_V70del_Dropout | K417N | N439K_Dropout | P681H | S477N | T478K | T95I | Y144del_Composite
PV45482	12/14/21	EPI_ISL_7908094	BA.1	D614G Detected. Omicron (B.1.1.529) Detected.	A701V | D614G | E484-K/Q_Dropout | H69_V70del_Dropout | K417N | N439K_Dropout | P681H | S477N | T478K | T95I
PV45433	12/14/21	EPI_ISL_7908016	BA.1	D614G Detected. Omicron (B.1.1.529) Detected.	A701V | D614G | H69_V70del_Dropout | K417N | N439K_Dropout | P681H | S477N | T478K | T95I | Y144del_Composite
PV45432	12/14/21	EPI_ISL_7908072	BA.1.1	D614G Detected. Omicron (B.1.1.529) Detected.	D614G | H69_V70del_Dropout | K417N | N439K_Dropout | P681H | S477N | T478K | T95I | Y144del_Composite
PV45484	12/14/21	EPI_ISL_7908047	BA.1.1	D614G Detected. Omicron (B.1.1.529) Detected.	D614G | H69_V70del_Dropout | K417N | N439K_Dropout | P681H | S477N | T478K | T95I | Y144del_Composite

PV45438	12/14/21	EPI_ISL_7908085	BA.1	D614G Detected. Omicron (B.1.1.529) Detected.	D614G | H69_V70del_Dropout | K417N | N439K_Dropout | P681H | S477N | T478K | T95I | Y144del_Composite
PV45479	12/14/21	EPI_ISL_7908046	BA.1	D614G Detected. Omicron (B.1.1.529) Detected.	D614G | H69_V70del_Dropout | K417N | N439K_Dropout | P681H | S477N | T478K | T95I | Y144del_Composite
PV45463	12/14/21	EPI_ISL_7908044	BA.1	D614G Detected. Omicron (B.1.1.529) Detected.	D614G | H69_V70del_Dropout | K417N | N439K_Dropout | P681H | S477N | T478K | T95I | Y144del_Composite
PV45510	12/14/21	EPI_ISL_7908049	BA.1	D614G Detected.	D614G | E484-K/Q_Dropout | H69_V70del_Dropout | N439K_Dropout | S477N | T478K | T95I
PV45514	12/14/21	EPI_ISL_7908050	BA.1.1	D614G Detected. Omicron (B.1.1.529) Detected.	D614G | H69_V70del_Dropout | K417N | P681H | S477N | T478K | T95I | Y144del_Composite
PV45440	12/14/21	EPI_ISL_7908002	BA.1	D614G Detected. Omicron (B.1.1.529) Detected.	D614G | H69_V70del_Dropout | K417N | N439K_Dropout | P681H | S477N | T478K | T95I | Y144del_Composite
PV45505	12/14/21	EPI_ISL_7908063	BA.1	D614G Detected. Omicron (B.1.1.529) Detected.	D614G | H69_V70del_Dropout | K417N | N439K_Dropout | P681H | S477N | T478K | T95I | Y144del_Composite
PV45500	12/14/21	EPI_ISL_7908075	BA.1	D614G Detected. Omicron (B.1.1.529) Detected.	D614G | H69_V70del | K417N | N439K_Dropout | P681H | S477N | T478K | T95I | Y144del_Composite
PV45480	12/14/21	EPI_ISL_7908092	BA.1	D614G Detected. Omicron (B.1.1.529) Detected.	D614G | H69_V70del_Dropout | K417N | N439K_Dropout | P681H | S477N | T478K | T95I | Y144del_Composite
PV45449	12/14/21	EPI_ISL_7908073	BA.1	D614G Detected. Omicron (B.1.1.529) Detected.	D614G | H69_V70del_Dropout | K417N | N439K_Dropout | P681H | S477N | T478K | T95I | Y144del_Composite
PV45492	12/14/21	EPI_ISL_7908048	BA.1	D614G Detected. Omicron (B.1.1.529) Detected.	A701V | D614G | H69_V70del_Dropout | K417N | N439K_Dropout | P681H | S477N | T478K | T95I | Y144del_Composite
PV45494	12/14/21	EPI_ISL_7908057	BA.1	D614G Detected. Omicron (B.1.1.529) Detected.	D614G | H69_V70del_Dropout | K417N | N439K_Dropout | P681H | S477N | T478K | T95I | Y144del_Composite
PV45437	12/14/21	EPI_ISL_7908069	BA.1	D614G Detected. Omicron (B.1.1.529) Detected.	D614G | H69_V70del_Dropout | K417N | N439K_Dropout | P681H | S477N | T478K | T95I | Y144del_Composite
PV48991	12/19/21	EPI_ISL_11178972	AY.44	D614G Detected. Delta (B.1.617.2) Detected.	D614G | L452R | P681R | T19R | T478K

PV47876	12/20/21	EPI_ISL_11178970	BA.1	D614G Detected.	D614G | H69_V70del_Dropout | N439K_Dropout | S477N | T478K | T95I
PV48993	12/21/21	EPI_ISL_11178973	AY.39.1	D614G Detected. Delta (B.1.617.2) Detected.	D614G | H69_V70del | L452R | P681R | T19R | T478K | T95I
PV48978	12/25/21	EPI_ISL_11178971	AY.44	D614G Detected. Delta (B.1.617.2) Detected.	D614G | L452R | P681R | T19R | T478K
PV49009	12/27/21	EPI_ISL_11178974	AY.109	D614G Detected. Delta (B.1.617.2) Detected.	D614G | H69_V70del | L452R | P681R | T19R | T478K | T95I
PV49041	12/27/21	EPI_ISL_11178975	AY.3.3	D614G Detected. Delta (B.1.617.2) Detected.	D614G | H69_V70del | L452R | P681R | T19R | T478K
PV50567	1/3/22	EPI_ISL_10831379	BA.1	D614G Detected. Omicron (B.1.1.529) Detected.	D614G | H69_V70del_Dropout | K417N | N439K_Dropout | P681H | S477N | T478K | T95I | Y144del_Composite
PV50565	1/3/22	EPI_ISL_10831377	BA.1	D614G Detected. Omicron (B.1.1.529) Detected.	D614G | H69_V70del_Dropout | K417N | N439K_Dropout | P681H | S477N | T478K | T95I | Y144del_Composite
PV50564	1/3/22	EPI_ISL_10831376	BA.1.1	D614G Detected. Omicron (B.1.1.529) Detected.	D614G | H69_V70del_Dropout | K417N | N439K_Dropout | P681H | S477N | T478K | T95I | Y144del_Composite
PV50568	1/3/22	EPI_ISL_10831380	BA.1.1	D614G Detected.	D614G | E484-K/Q_Dropout | H69_V70del_Dropout | N439K_Dropout | S477N | T478K | T95I
PV50450	1/4/22	EPI_ISL_10831369	AY.43	D614G Detected. Delta (B.1.617.2) Detected.	D614G | L452R | P681R | T19R | T478K
PV50577	1/4/22	EPI_ISL_10831389	BA.1.1	D614G Detected. Omicron (B.1.1.529) Detected.	D614G | H69_V70del_Dropout | K417N | N439K_Dropout | P681H | S477N | T478K | T95I | Y144del_Composite
PV50569	1/4/22	EPI_ISL_10831381	BA.1.1	D614G Detected. Omicron (B.1.1.529) Detected.	D614G | H69_V70del_Dropout | K417N | N439K_Dropout | P681H | S477N | T478K | T95I | Y144del_Composite
PV50571	1/4/22	EPI_ISL_10831383	BA.1.1	D614G Detected. Omicron (B.1.1.529) Detected.	D614G | H69_V70del_Dropout | K417N | N439K_Dropout | P681H | S477N | T478K | T95I | Y144del_Composite
PV50451	1/4/22	EPI_ISL_10831370	BA.1	D614G Detected. Omicron (B.1.1.529) Detected.	D614G | H69_V70del_Dropout | K417N | N439K_Dropout | P681H | S477N | T478K | T95I | Y144del_Composite
PV50489	1/4/22	EPI_ISL_10831372	BA.1.1	D614G Detected. Omicron (B.1.1.529) Detected.	D614G | H69_V70del_Dropout | K417N | N439K_Dropout | P681H | S477N | T478K | T95I | Y144del_Composite
PV50615	1/4/22	EPI_ISL_10831396	BA.1.1	D614G Detected. Omicron (B.1.1.529) Detected.	D614G | H69_V70del_Dropout | K417N | N439K_Dropout | P681H | S477N | T478K | T95I | Y144del_Composite

PV50488	1/4/22	Mixed	BA.1.1	D614G Detected. Omicron (B.1.1.529) Detected.	D614G | H69_V70del_Dropout | K417N | N439K_Dropout | P681H | S477N | T478K | T95I | Y144del_Composite
PV50574	1/4/22	EPI_ISL_10831386	BA.1.1	D614G Detected. Omicron (B.1.1.529) Detected.	D614G | H69_V70del_Dropout | K417N | N439K_Dropout | P681H | S477N | T478K | T95I | Y144del_Composite
PV50570	1/4/22	EPI_ISL_10831382	BA.1	D614G Detected. Omicron (B.1.1.529) Detected.	A701V | D614G | H69_V70del_Dropout | K417N | N439K_Dropout | P681H | S477N | T478K | T95I | Y144del_Composite
PV50576	1/4/22	EPI_ISL_10831388	BA.1	D614G Detected. Omicron (B.1.1.529) Detected.	A701V | D614G | H69_V70del_Dropout | K417N | N439K_Dropout | P681H | S477N | T478K | T95I | Y144del_Composite
PV50501	1/4/22	EPI_ISL_10831375	BA.1.1	D614G Detected. Omicron (B.1.1.529) Detected.	D614G | H69_V70del_Dropout | K417N | N439K_Dropout | P681H | S477N | T478K | T95I | Y144del_Composite
PV50575	1/4/22	EPI_ISL_10831387	BA.1	D614G Detected. Omicron (B.1.1.529) Detected.	A701V | D614G | H69_V70del_Dropout | K417N | N439K_Dropout | P681H | S477N | T478K | T95I | Y144del_Composite
PV50572	1/4/22	EPI_ISL_10831384	BA.1.1	D614G Detected. Omicron (B.1.1.529) Detected.	D614G | H69_V70del_Dropout | K417N | N439K_Dropout | P681H | S477N | T478K | T95I | Y144del_Composite
PV50566	1/4/22	EPI_ISL_10831378	BA.1	D614G Detected. Omicron (B.1.1.529) Detected.	D614G | H69_V70del_Dropout | K417N | N439K_Dropout | P681H | S477N | T478K | T95I | Y144del_Composite
PV50573	1/4/22	EPI_ISL_10831385	BA.1	D614G Detected. Omicron (B.1.1.529) Detected.	D614G | H69_V70del_Dropout | K417N | N439K_Dropout | P681H | S477N | T478K | T95I | Y144del_Composite
PV50579	1/4/22	EPI_ISL_10831391	BA.1.1	D614G Detected. Omicron (B.1.1.529) Detected.	D614G | H69_V70del_Dropout | K417N | N439K_Dropout | P681H | S477N | T478K | T95I | Y144del_Composite
PV50578	1/4/22	EPI_ISL_10831390	BA.1.1	D614G Detected. Omicron (B.1.1.529) Detected.	D614G | H69_V70del_Dropout | K417N | N439K_Dropout | P681H | S477N | T478K | T95I | Y144del_Composite
PV50491	1/4/22	EPI_ISL_10831374	BA.1	D614G Detected. Omicron (B.1.1.529) Detected.	D614G | H69_V70del_Dropout | K417N | N439K_Dropout | P681H | S477N | T478K | T95I
PV50490	1/4/22	EPI_ISL_10831373	BA.1	D614G Detected. Omicron (B.1.1.529) Detected.	D614G | H69_V70del_Dropout | K417N | N439K_Dropout | P681H | S477N | T478K | T95I | Y144del_Composite

PV50485	1/5/22	EPI_ISL_10831371	BA.1	D614G Detected. Omicron (B.1.1.529) Detected.	D614G | H69_V70del_Dropout | K417N | N439K_Dropout | P681H | S477N | T478K | T95I | Y144del_Composite
PV50446	1/5/22	EPI_ISL_10831365	BA.1.1	D614G Detected. Omicron (B.1.1.529) Detected.	D614G | H69_V70del_Dropout | K417N | N439K_Dropout | P681H | S477N | T478K | T95I | Y144del_Composite
PV50448	1/5/22	EPI_ISL_10831367	BA.1	D614G Detected. Omicron (B.1.1.529) Detected.	D614G | H69_V70del | K417N | N439K_Dropout | P681H | S477N | T478K | T95I | Y144del_Composite
PV50449	1/5/22	EPI_ISL_10831368	BA.1	D614G Detected. Omicron (B.1.1.529) Detected.	D614G | H69_V70del_Dropout | K417N | N439K_Dropout | P681H | S477N | T478K | T95I | Y144del_Composite
PV50444	1/5/22	EPI_ISL_10831364	BA.1	D614G Detected. Omicron (B.1.1.529) Detected.	D614G | H69_V70del_Dropout | K417N | N439K_Dropout | P681H | S477N | T478K | T95I | Y144del_Composite
PV50429	1/10/22	EPI_ISL_10831356	BA.1	D614G Detected. Omicron (B.1.1.529) Detected.	D614G | H69_V70del_Dropout | K417N | N439K_Dropout | P681H | S477N | T478K | T95I | Y144del_Composite
PV50436	1/10/22	EPI_ISL_10831359	BA.1.1	D614G Detected. Omicron (B.1.1.529) Detected.	D614G | H69_V70del_Dropout | K417N | N439K_Dropout | P681H | S477N | T478K | T95I | Y144del_Composite
PV50430	1/10/22	EPI_ISL_10831357	BA.1.1	D614G Detected. Omicron (B.1.1.529) Detected.	D614G | H69_V70del_Dropout | K417N | N439K_Dropout | P681H | S477N | T478K | T95I | Y144del_Composite
PV50608	1/10/22	EPI_ISL_10831394	BA.1.1	D614G Detected. Omicron (B.1.1.529) Detected.	D614G | H69_V70del_Dropout | K417N | N439K_Dropout | P681H | S477N | T478K | T95I | Y144del_Composite
PV50609	1/11/22	EPI_ISL_10831395	BA.1	D614G Detected. Omicron (B.1.1.529) Detected.	D614G | H69_V70del_Dropout | K417N | N439K_Dropout | P681H | S477N | T478K | T95I | Y144del_Composite
PV50433	1/11/22	EPI_ISL_10831358	BA.1	D614G Detected. Omicron (B.1.1.529) Detected.	D614G | K417N | N439K_Dropout | P681H | S477N | T478K | T95I | Y144del_Composite
PV50438	1/11/22	EPI_ISL_10831360	BA.1.1	D614G Detected. Omicron (B.1.1.529) Detected.	D614G | H69_V70del_Dropout | K417N | N439K_Dropout | P681H | S477N | T478K | T95I | Y144del_Composite
PV50441	1/11/22	EPI_ISL_10831362	BA.1	D614G Detected. Omicron (B.1.1.529) Detected.	D614G | H69_V70del_Dropout | K417N | N439K_Dropout | P681H | S477N | T478K | T95I | Y144del_Composite
PV50439	1/11/22	EPI_ISL_10831361	BA.1.1	D614G Detected. Omicron (B.1.1.529) Detected.	D614G | H69_V70del_Dropout | K417N | N439K_Dropout |

					P681H | S477N | T478K | T95I | Y144del_Composite
PV50607	1/11/22	EPI_ISL_10831393	BA.1.1	D614G Detected. Omicron (B.1.1.529) Detected.	D614G | K417N | N439K_Dropout | P681H | S477N | T478K | T95I
PV50605	1/11/22	EPI_ISL_10831392	BA.1.1	D614G Detected. Omicron (B.1.1.529) Detected.	D614G | H69_V70del_Dropout | K417N | N439K_Dropout | P681H | S477N | T478K | T95I | Y144del_Composite
PV51975	1/24/22	EPI_ISL_11178976	BA.2	D614G Detected.	D614G | K417N | N439K_Dropout | N501Y | P681H | S477N | T478K
PV55589	2/1/22	EPI_ISL_11178988	BA.2	D614G Detected.	D614G | K417N | N439K_Dropout | N501Y | P681H | S477N | T478K
PV55575	2/1/22	EPI_ISL_11178987	BA.2	D614G Detected.	D614G | K417N | N439K_Dropout | N501Y | P681H | S477N | T478K
PV56004	2/15/22	EPI_ISL_11179017	BA.2	D614G Detected.	D614G | N439K_Dropout | N501Y | P681H | S477N | T478K
PV56011	2/16/22	EPI_ISL_11179020	BA.2	D614G Detected.	D614G | K417N | N439K_Dropout | N501Y | P681H | S477N | T478K
PV56107	2/22/22	EPI_ISL_11179051	BA.2	D614G Detected.	D614G | K417N | N439K_Dropout | N501Y | P681H | S477N | T478K
PV56159	2/24/22	EPI_ISL_11179065	BA.2	D614G Detected.	D614G | K417N | N439K_Dropout | N501Y | P681H | S477N | T478K
PV56250	2/25/22	EPI_ISL_11179073	BA.2	D614G Detected.	D614G | K417N | N439K_Dropout | N501Y | P681H | S477N | T478K
PV56302	3/2/22	EPI_ISL_11179077	BA.2	D614G Detected.	D614G | N439K_Dropout | N501Y | P681H | S477N | T478K

[a] GISAID accession IDs are indicated for specimens with single variant consensus genomes; mixed assemblies are not deposited in GISAID.

[b] Variant ID result from variant report output file.

[c] SARS-CoV-2 amino acid polymorphisms separated by bars ("|") per the variant report output file. Native amino acid calls are not listed if detected.

Table S5. Predictive values for panel variant calls

Variant	Number of Specimens [a]	PPV (95% CI)	NPV (95% CI)	P-value [b]
Omicron (BA.1)	79	1.000 (0.951-1.000)	0.984 (0.964-0.993)	<0.0001
Delta	110	0.991 (0.949-1.000)	0.986 (0.964-0.995)	<0.0001
Alpha	40	1.000 (0.912-1.000)	1.000 (0.989-1.000)	<0.0001
Beta	4	1.000 (0.439-1.000)	0.997 (0.986-1.000)	<0.0001
Gamma	14	0.933 (0.702-0.997)	1.000 (0.990-1.000)	<0.0001
Zeta	1	0 (0.000-0.204)	0.997 (0.985-1.000)	>0.999
Eta	7	1.000 (0.051-1.000)	0.985 (0.967-0.993)	0.0179
Iota	39	1.000 (0.908-1.000)	0.997 (0.984-1.000)	<0.0001
Epsilon	19	1.000 (0.832-1.000)	1.000 (0.990-1.000)	<0.0001
B.1.258	1	1.000 (0.051-1.000)	1.000 (0.990-1.000)	0.0026
D614G [c]	27	NA	NA	NA
Broad USA	5	1.000 (0.566-1.000)	1.000 (0.990-1.000)	<0.0001

[a] Number of specimens confirmed by WGS as the indicated variant.
[b] By Fisher's exact test.
[c] Agreement analyses were not performed as specimens that both (1) harbored the native D614 amino acid and (2) did not identify as any other variant on the panel were not recovered for testing. NA, not available.

Table S6. Predictive values for panel target calls

Target	Number of Specimens [a, b]	PPV (95% CI)	NPV (95% CI)	P-value [c]
L5F	42	1.000 (0.912-1.000)	0.994 (0.980-0.999)	<0.0001
S13I	19	1.000 (0.824-1.000)	0.997 (0.985-1.000)	<0.0001
L18F	15	0.790 (0.567-0.915)	1.000 (0.990-1.000)	<0.0001
T19R	110	0.991 (0.949-1.000)	0.986 (0.964-0.995)	<0.0001
H69_V70del	51	0.922 (0.815-0.969)	0.984 (0.960-0.994)	<0.0001
D80A	4	1.000 (0.439-1.000)	0.997 (0.986-1.000)	<0.0001
D80G	0	NA	NA	NA
T95I	159	0.924 (0.874-0.955)	0.996 (0.975-1.000)	<0.0001
Y144del	126	0.881 (0.813-0.927)	0.943 (0.909-0.965)	<0.0001
W152C	19	1.000 (0.832-1.000)	1.000 (0.990-1.000)	<0.0001
D215G	3	0	0.992 (0.978-0.998)	>0.9999
L242_244del	4	0.667 (0.119-0.983)	0.995 (0.981-0.999)	0.0002
D253G	39	0.927 (0.806-0.975)	0.997 (0.984-1.000)	<0.0001
K417N	92	0.988 (0.937-0.999)	0.977 (0.953-0.989)	<0.0001
K417T	14	0.824 (0.590-0.938)	1.000 (0.990-1.000)	<0.0001
N439K	1	1.000 (0.051-1.000)	1.000 (0.987-1.000)	0.0034
L452R	124	0.961 (0.912-0.983)	0.996 (0.979-1.000)	<0.0001
Y453F	0	NA	NA	NA
S477N	90	1.000 (0.959-1.000)	1.000 (0.987-1.000)	<0.0001
T478K	194	0.980 (0.949-0.992)	0.995 (0.971-1.000)	<0.0001
E484Q	0	NA	NA	NA
E484K	65	0.891 (0.791-0.946)	0.974 (0.951-0.987)	<0.0001
Q493K	0	NA	NA	NA
N501Y	158	0.963 (0.898-0.990)	0.744 (0.693-0.790)	<0.0001
N501Y (exclude BA.1)	79	0.963 (0.898-0.990)	1.000 (0.984-1.000)	<0.0001
N501T	0	NA	NA	NA
A570D	40	1.000 (0.912-1.000)	1.000 (0.989-1.000)	<0.0001
D614G	388	1.000 (0.990-1.000)	0 (0.000-0.822)	>0.9999
Q677H	7	0.700 (0.397-0.892)	1.000 (0.990-1.000)	<0.0001
Q677P	5	1.000 (0.566-1.000)	1.000 (0.990-1.000)	<0.0001

P681H	141	0.986 (0.949-0.997)	0.984 (0.960-0.994)	<0.0001
P681R	110	1.000 (0.964-1.000)	0.979 (0.955-0.990)	<0.0001
I692V	0	NA	NA	NA
A701V	49	0.925 (0.821-0.970)	1.000 (0.989-1.000)	<0.0001
T716I	40	1.000 (0.912-1.000)	1.000 (0.989-1.000)	<0.0001
S982A	40	1.000 (0.910-1.000)	0.997 (0.984-1.000)	<0.0001
K1191N	2	0.667 (0.119-0.983)	1.000 (0.990-1.000)	<0.0001

[a] Number of specimens that harbor the given target polymorphism by WGS.
[b] Analyses were not performed if specimens with the given target polymorphism by WGS were not recovered for testing. NA, not available.
[c] By Fisher's exact test.
